# Ancient dental calculus preserves signatures of biofilm succession and interindividual variation independent of dental pathology

**DOI:** 10.1093/pnasnexus/pgac148

**Published:** 2022-08-04

**Authors:** Irina M Velsko, Lena Semerau, Sarah A Inskip, Maite I García-Collado, Kirsten Ziesemer, Maria Serrano Ruber, Luis Benítez de Lugo Enrich, Jesús Manuel Molero García, David Gallego Valle, Ana Cristina Peña Ruiz, Domingo C Salazar-García, Menno L P Hoogland, Christina Warinner

**Affiliations:** Department of Archaeogenetics, Max Planck Institute for Evolutionary Anthropology, Leipzig 04103, Germany; Department of Archaeogenetics, Max Planck Institute for Evolutionary Anthropology, Leipzig 04103, Germany; Faculty of Biological Sciences, Friedrich Schiller University, Jena 07743, Germany; School of Archaeology and Ancient History, University of Leicester, University Road, Leicester LE1 7RH, UK; GIPYPAC, Department of Geography, Prehistory and Archaeology, University of the Basque Country, Leioa 48940, Spain; BioArCh, Department of Archaeology, University of York, York YO10 5NG, UK; University Library, Vrije Universiteit, Einsteinweg 2, Amsterdam 1081 HV, The Netherlands; School of Archaeology and Ancient History, University of Leicester, University Road, Leicester LE1 7RH, UK; Departmento de Prehistoria, Historia Antigua y Arqueología, Universidad Complutense de Madrid, Madrid 28040, Spain; Facultad de Letras, Universidad de Castilla-La Mancha, Ciudad Real 13004, Spain; Facultad de Letras, Universidad de Castilla-La Mancha, Ciudad Real 13004, Spain; Facultad de Bellas Artes, Universidad de Castilla-La Mancha, Cuenca 13004, Spain; Departament de Prehistòria, Historia i Arqueología, Universitat de València, València 46010, Spain; Department of Geological Sciences, University of Cape Town, Rondebosch 7701, South Africa; Faculty of Archaeology, Leiden University, Einsteinweg, Leiden 2333 CC, The Netherlands; Department of Archaeogenetics, Max Planck Institute for Evolutionary Anthropology, Leipzig 04103, Germany; Faculty of Biological Sciences, Friedrich Schiller University, Jena 07743, Germany; Department of Anthropology, Harvard University, Cambridge, MA 02138, USA

**Keywords:** ancient DNA, dental calculus, metagenomics, tobacco, smoking

## Abstract

Dental calculus preserves oral microbes, enabling comparative studies of the oral microbiome and health through time. However, small sample sizes and limited dental health metadata have hindered health-focused investigations to date. Here, we investigate the relationship between tobacco pipe smoking and dental calculus microbiomes. Dental calculus from 75 individuals from the 19th century Middenbeemster skeletal collection (Netherlands) were analyzed by metagenomics. Demographic and dental health parameters were systematically recorded, including the presence/number of pipe notches. Comparative data sets from European populations before and after the introduction of tobacco were also analyzed. Calculus species profiles were compared with oral pathology to examine associations between microbiome community, smoking behavior, and oral health status. The Middenbeemster individuals exhibited relatively poor oral health, with a high prevalence of periodontal disease, caries, heavy calculus deposits, and antemortem tooth loss. No associations between pipe notches and dental pathologies, or microbial species composition, were found. Calculus samples before and after the introduction of tobacco showed highly similar species profiles. Observed interindividual microbiome differences were consistent with previously described variation in human populations from the Upper Paleolithic to the present. Dental calculus may not preserve microbial indicators of health and disease status as distinctly as dental plaque.

Significance Statement In contrast to strong correlations observed between dental plaque microbiomes and oral health in people living today, no associations between historic calculus microbiome profiles and oral health metrics were detected in a large archaeological population. Instead, historic calculus species profiles fall along a gradient, wherein samples at one end are enriched in oxygen-tolerant species, and those at the other are enriched in oxygen-sensitive species. This indicates calculus microbiomes are primarily shaped by individual-specific biofilm developmental processes that are independent of host health. By studying host–microbiome coevolution with a hologenomic approach integrating osteological dental health data in archaeological populations with ancient metagenomics, we observe that the strongest factors shaping microbiome composition in living populations differ from those functioning on evolutionary-timescales.

## Introduction

Dental calculus is a mineralized form of dental plaque that forms on the surface of teeth during life and persists in the archaeological record. Diverse microremains and biomolecules, including DNA, protein, and metabolites, are preserved within ancient dental calculus (1–7) and can be used to study oral microbial ecology and evolution through time (8–10), as well as provide evidence of past human activities (11). The majority of biomolecules present in calculus derive from dental plaque bacteria, and there is great interest in determining the feasibility of using these microbes to indirectly trace evidence of human behavioral or lifestyle changes and their impact on health through deep time. Certain past activities, such as the rise and spread of tobacco smoking during the European Colonial period, can be difficult to detect directly but likely had important health consequences. Examining dental plaque communities via dental calculus may enable the detection of tobacco use and its growing impact on oral health, but to date this topic has not been extensively explored.

**Fig. 1.**
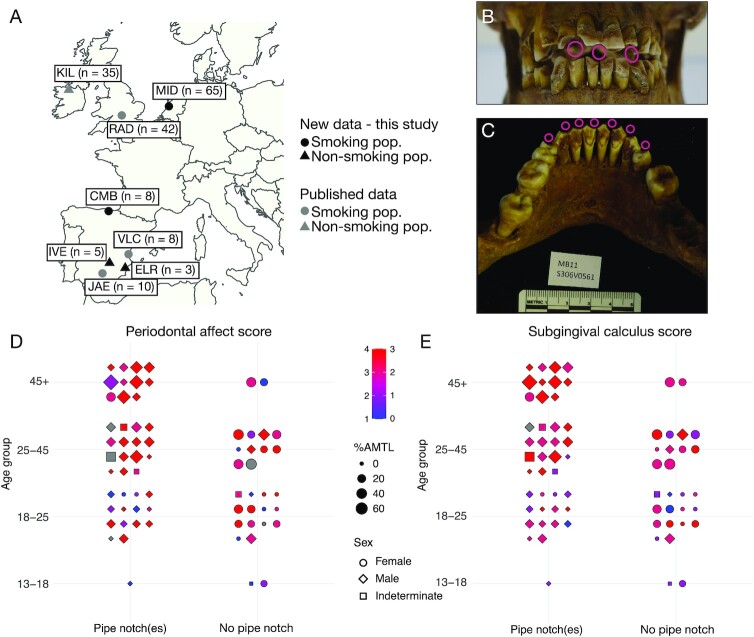
The Middenbeemster collection. (A) Map of sites included in the study. (B) Male individual from Middenbeemster with three prominent pipe notches in the anterior dentition indicated by hollow pink dots. (C) Mandible of a male individual S306V0561 with at least seven pipe notches on the anterior dentition, indicated by hollow pink dots. (D) and (E) Dental pathology of Middenbeemster individuals in relation to age, sex, antemortem tooth loss (AMTL), and presence of pipe notches, points colored by (D) periodontal affect score (1 to 4), or (E) subgingival calculus score (0 to 3). Gray indicates no data. Sample sites: CMB—Convento de los Mercedarios de Burtzeña; ELR—El Raval; IVE—Iglesia de la Virgen de la Estrella; JAE—Jaen; KIL—Kilteasheen; MID—Middenbeemster; RAD—Radcliffe; and VLC—Valencia. Photo credit: Sarah A. Inskip.

Tobacco was introduced to Europe from the Americas at the turn of the 15th century, initially as a medical remedy (12). However, by the late 16th century, tobacco smoking had become a popular social and leisure activity, particularly in Western Europe (12–14). Archaeologically, it has been possible to detect pipe smoking through the identification of so-called dental “pipe notches.” Pipe notches are areas of dental abrasion caused by habitually clenching a clay pipe between the anterior teeth (Figure 1B and C; Figure S1). Multiple studies of these features in 17th to 19th century European populations have shown that pipe smoking was a predominantly male activity, which varied in popularity over time but increasingly became linked to lower socioeconomic status in the 18th and 19th century (15–19). These trends correspond to historical records about tobacco use and smoking in England and the Netherlands (12, 20, 21).

Studies of present-day tobacco users have reported a negative relationship between tobacco use and oral health (22–24). Tobacco users have been shown to have more severe dental and periodontal pathologies, including caries and tooth loss (25, 26), periodontal disease (27–29), and calculus accumulation (22, 29–31). Chemicals inhaled in tobacco smoke appear to affect oral microbes and promote a more pathogenic community, such that multiple studies have found that smokers have distinct dental plaque microbial communities compared to nonsmokers (32–36). However, the extent to which these differences are also present and persist in dental calculus, which represents a more mature biofilm than dental plaque (4), is not known. Potentially, dental calculus may preserve these distinctions, and if so, would offer the possibility of identifying heavy smokers in the past, including in cases where abrasive clay pipes were not used.

Here, we investigated the dental calculus microbial communities associated with pipe smoking in historic European populations, particularly focusing on 19th century individuals from Middenbeemster (MID), the Netherlands (Figure 1A). We first performed an in-depth osteological assessment of dental pathology in the MID dataset to understand the distribution of dental disease in this population. As dental pathology influences the microbial profile of dental plaque in living populations, we aimed to explore whether the microbial profiles of dental calculus in MID individuals was similarly associated with pathology. We focused particularly on the presence of pipe notches in anterior teeth, which indicate heavy pipe use during life, because tobacco use is associated with altered species profiles in living populations (32–36). We then explored the dental calculus microbial profiles of the MID population along with the contemporaneous Convento de los Mercedarios de Burtzeña (CMB) population from Spain, who also have pipe notches in their dentition. Next, we broadened our study to examine whether dental calculus profiles differ between populations living during distinct European historical periods preceding (Medieval), contemporary with (Industrial), and postceding (present-day Modern) the introduction of tobacco to Europe, regardless of oral health. These included previously published data from Medieval Ireland (KIL) (37), Industrial-era England (RAD) (4), and present-day modern Spain (JAE and VLC) (4, 8), as well as two new datasets generated for this study from medieval Spain (ELR and IVE; Figure 1A).

Contrary to expectations, we found that there are minimal differences in the dental calculus microbial structure of individuals with evidence of heavy pipe smoking (pipe notches) and those without, despite evidence for differences in skeletal markers of oral disease between those groups. Moreover, we find that through comparative analysis with additional Medieval, Industrial, and Modern (present-day) individuals, this pattern holds more broadly, with no discernible differences in overall microbial community profiles between individuals who lived before and after the introduction of tobacco to Europe.

## Results

### Dentition

#### Demography and pipe use

Dental calculus was collected from a total of 75 individuals in the MID collection, of which 69 had observable anterior dentition, which allowed observation of presence or absence of pipe notches. These individuals consisted of 39 males, 25 females, and five individuals of unknown sex. A total of 66 of individuals could be aged (Table 1). Of the 25 females, 24 could be attributed an age, while for the 39 males, 38 could be provided an age estimate. In general, there are proportionally more females in the young category, and more males in the old category, which needs to be considered when assessing oral pathology.

**Table 1.** Age and sex distribution of individuals in the study sample.

Age (years)	Number males^1^	Number females^1^	Number unknown sex^1^
Young (18 to 25)	14 (21.2%)	13 (19.7%)	1 (1.5%)
Middle (25 to 45)	14 (21.2%)	8 (12.1%)	0
Old (> 45)	10 (15.2%)	3 (4.5%)	3 (4.5%)
Unknown	1 (1.5%)	1 (1.5%)	1 (1.5%)
Total	39	25	5

^1^Percent indicates % of total aged individuals (38).

The dentition was sufficiently intact to score the presence or absence of pipe notches in 70 individuals (Table S2; Figure 1B). We observed a strong relationship between biological sex and pipe smoking, with most individuals bearing pipe notches being male. Only 3 of 25 females had pipe notches (12%). A total of two of these women had one notch, and the other had two. Conversely, most men exhibited pipe notches (88%; 35/40). Of the five men who did not have a notch, two of these are young males and three are middle-age males. Of those with notches, 75% had at least two notches. The maximum number of notches observed among men was seven (Figure 1C). The pattern of pipe notches in males suggests that habitual pipe smoking started from a young age. Furthermore, based on this data, pipe smokers were slightly older on average than nonsmokers.

### Oral pathologies by pipe use status

While there was no difference in the incidence of caries between individuals with and without pipe notches, individuals without pipe notches had a higher percentage of teeth with caries (26%) compared to pipe-users (15.1%; *t* = 2.1194, df = 66, *P* = 0.038), and more nonusers had gross caries (50%) than pipe-users (27.5%). However, there was a trend for more of the pipe users to have lost teeth antemortem (80.5%) than nonusers (67.9%), possibly confounding our observations (Table S3).

Early stages of periodontal disease (stage 2) was observable in almost all individuals. As such, we assessed whether there were differences in more severe stages of periodontal disease between pipe-users and nonusers (stages 3 and 4). More pipe-users had periodontal disease at stage 3 or 4 (87.8%) than nonusers (64.3%), a difference that was statistically significant by chi-squared test (*P* = 0.035, *n* = 69). In terms of periapical lesions, more pipe users were affected (45%) compared to nonusers (35.7%), and had more positions affected (3.4) compared to nonusers (2.4). Additionally, dental calculus was almost ubiquitous in the sample collection, so we assessed how many teeth were affected and the general level of severity in the mouth. Pipe users had a greater proportion of teeth with dental calculus (73.5%) compared to nonusers (58.6%), and had a greater build-up of both supra- and subgingival calculus.

To better understand patterns of oral pathologies, we assessed the relationship between each condition and age. There was a positive relationship between prevalence and age for all oral pathologies with the exception of caries and gross caries. Caries prevalence was similar in all age groups, while gross caries became less common with age. While this may seem counterintuitive, caries is a leading cause of tooth loss, and AMTL prevalence was highest in the oldest age category. The lower prevalence of caries, especially of gross caries, in the oldest category is likely due to the high degree of observed tooth loss.

Figure 1(D) and (E) graphically depict the severity of three oral pathologies that have been linked to smoking in present-day modern populations, including periodontal disease, antemortem tooth loss (AMTL) and subgingival calculus (39–43), by age and sex. For all three oral pathologies, middle-age and old-age adults tended to have more severe manifestations of the conditions, with nearly all individuals in the 45+ years category having periodontal disease scores of 3 or 4, calculus scores of 2 or 3, and high rates of tooth loss (mean 28% of teeth per individual, compared to mean 8.8% for all other age groups). As such, it is evident that there is a strong positive relationship between the severity of oral pathologies and age but not sex (Figure 1D and E).

### Calculus preservation assessment

To explore whether microbial signatures of smoking could be detected from 18th century Europe, we analyzed dental calculus samples from MID, the Netherlands, and CMB, Spain. Dental calculus microbial community preservation was high at both sites (Figures S2 and S3), with all but three MID samples passing quality control thresholds for preservation. A total of eight MID samples did not have sufficient metadata for further comparisons and were excluded from all microbiome analysis. After filtering for preservation and metadata completeness, 73 samples were used in downstream analyses (MID = 65, CMB = 8).

### Microbial species profile differences

**Fig. 2.**
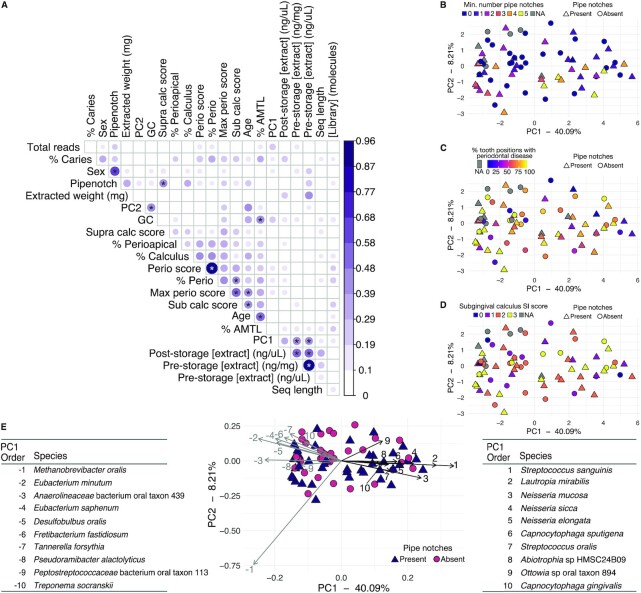
Microbial community diversity and correlations with oral pathology and laboratory work metadata. (A) CC for MID samples between metadata categories and principal components loadings. Significance tests were performed with a Pearson correlation test. The size and color of the dots corresponds to the CC value, which does not determine the direction of the correlation (positive or negative. Hence, all CC values are positive). Correlations ≥ 0.4 have significance indicated with stars. * *P* ≤ 0.001. (B)–(E) PCA based on species composition of MID and CMB samples colored by (B) Minimum number of pipe notches. (C) Percent of tooth positions with periodontal disease. (D) Subgingival calculus SI score. Samples from CMB are colored gray in (B)–(D) because due to the fragmented nature of the skeletons, the same metadata could not be collected (see Figure S1). (E) PCA based on species composition of MID and CMB samples colored by pipe notch presence, including a bi-plot indicating the loadings of 10 species with strongest positive and negative PC1 loadings, with the species indicated by number corresponding to the strength of loading (1/−1 is strongest, 10/−10 is weakest). The species are listed in tables to the left and right of the plot, ordered by decreasing strength of the loading. Metadata shown in (A): extracted weight (mg)—weight of calculus used in extraction; PC2–PC2 loadings; GC—library average GC content; % caries—% of teeth with caries; Sex—estimated biological sex; Pipenotch—pipe notch present; Seq length—library average sequence length; [Library] (molecules)—total DNA molecules in the library (x 106); Supra calc score—subragingival calculus SI score; % perioapical—% of teeth with perioapical lesions; % calculus—% of teeth with calculus; Perio score—average periodontitis score; % perio—% of teeth with periodontal disease; % AMTL—% of teeth lost ante-mortem; Max perio score—maximum periodontitis score; Sub calc score—subgingival calculus SI score; poststorage [extract] (ng/uL)—extract DNA concentration after storage; prestorage [extract] (ng/mg)—extract DNA concentration directly after extraction; prestorage [extract] (ng/uL)—extract DNA concentration directly after extraction; total reads—total reads in the library after quality-trimming and merging; and PC1–PC1 loadings.

We first wanted to determine if there are broadly discernable differences in calculus species profiles related to smoking evidence within the MID and CMB populations. To compare microbial profile differences related to smoking status, individuals were classified as heavy smokers if their dentition showed one or more pipe notches, or light/nonsmokers if there was no sign of pipe notches in their dentition. Beta-diversity analysis was performed with a principal components analysis (PCA) to compare species profiles. Canonical correlation (CC) analysis was used to assess correlations between sample metadata, including oral pathology, laboratory metrics, and sequencing analysis, as well as between sample metadata and principal component loadings (Figure 2A).

Strong correlations were found between sex and the presence of pipe notches, and between individual age at death and the following oral pathologies: % AMTL, % of teeth with periodontal disease, maximum periodontal disease score, and subgingival calculus score. PC1 loadings were found to be correlated with extracted DNA concentration, while PC2 loadings were correlated with average library GC content. However, no clustering with respect to microbial community composition and evidence of smoking status in PCA was observed or found with PERMANOVA testing (Table S9), such as minimum number of pipe notches (Figure 2B, R2 = 0.014, F = 0.88, *P* = 0.439), or periodontal health, such as % of tooth positions with periodontal disease (Figure 2C, R2 = 0.021, F = 1.3, *P* = 0.198), subgingival calculus score (Figure 2D, R2 = 0.018, F = 1.14, *P* = 0.282), or others (Figure S5). This suggests that these health metrics are not shaping the species profiles in this dataset, and other factors may be involved. Additionally, although most samples were pooled from multiple teeth, we did not find associations between microbial profile and the location of the tooth from which the calculus was collected (anterior or posterior; Table S9 and Figure S6), which supports recent findings that microbial profiles of calculus, unlike those of dental plaque, are not strongly shaped by tooth location (44).

To understand which species were driving the sample plotting patterns, we examined the top 10 species with the strongest positive and negative loadings in PC1 (Figure 2E). We found the species separating samples along PC1 have different environmental niches. The top 10 species with strongest negative loadings are largely anaerobic taxa that are dominant in mature oral biofilms, including those in the genera *Methanobrevibacter, Eubacterium, Desulfobulbus, Fretibacterium*, and *Tannerella*. In contrast, the top 10 species with strongest positive loadings are largely aerobic or facultative taxa that grow well in the presence of oxygen and are dominant in early dental biofilm formation, including those in the genera *Streptococcus, Neisseria*, and *Capnocytophaga*. The samples with high positive PC1 loadings, indicating a strong presence of oxygen-tolerant, early colonizer taxa, also show a higher proportion of “plaque” in the SourceTracker plots (Figure S3), supporting that they have a species profile that appears to have calcified at an earlier stage of development.

### Microbial functional profile

**Fig. 3.**
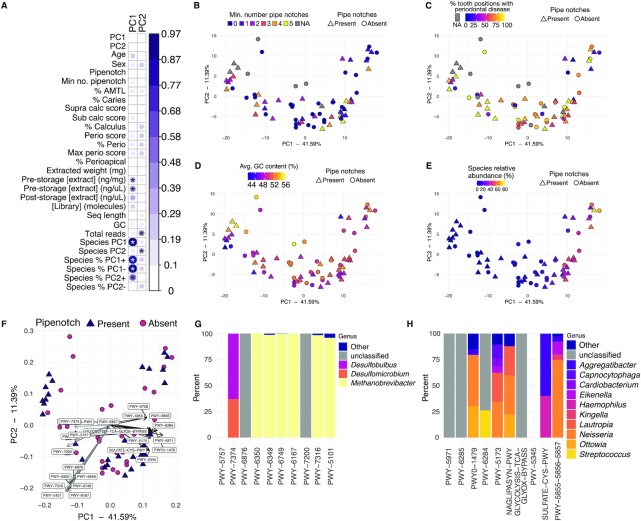
Metabolic pathway analysis and correlations. (A) CC for MID and CMB samples between metadata categories and principal component loadings for a PCA based on pathway abundance. Significance tests were performed with a Pearson correlation test. The size and color of the dots corresponds to the CC value, which does not determine the direction of the correlation (positive or negative. Hence, all CC values are positive). Correlations ≥ 0.4 have significance indicated with stars. * *P* ≤ 0.001. (B)–(E) PCA plot based on pathway abundance in MID and CMB samples colored by (B) minimum number of pipe notches, (C) percent of teeth with periodontal disease, (D) average GC content (%), and (E) relative abundance of the 10 species with strongest PC1 positive loadings from the species PCA in Figure 2(E). In (C) samples from CMB are colored gray because due to the fragmented nature of the skeletons, the same metadata could not be collected (see Figure S1). (F) PCA biplot showing the 20 pathways with strongest loadings in PC1 (10 highest positive loadings and 10 highest negative loadings). (G) and (H) show the % of each pathway contributed by species in the indicated genera. (G) A total of 10 pathways with strongest positive PC1 loadings. (H) A total of 10 pathways with strongest negative PC1 loadings. PWY-5855, PWY-5856, and PWY-5857 all have the same PC1 loading, and are contributed by the same proportions of the same species. All genera for which the total contribution was < 5% are grouped together as Other. The empty places for PWY-5757 in (D) and PWY-5345 in (G) indicate that HUMAnN3 was not able to attribute these pathways to specific species. Metadata shown in (A) PC1–PC1 loadings; PC2–PC2 loadings; age—estimated age at death; sex—estimated biological sex; pipenotch—one or more pipe notches present; min no. pipenotch—minimum number of pipe notches; % AMTL—% of teeth lost ante-mortem; % caries—% of teeth with caries; supra calc score—subragingival calculus SI score; sub calc score—subgingival calculus SI score; % calculus—% of teeth with calculus; perio score—average periodontitis score; % perio—% of teeth with periodontal disease; max perio score—maximum periodontitis score; % perioapical—% of teeth with perioapical lesions; extracted weight (mg)—weight of calculus used in extraction; prestorage [extract] (ng/mg)—extract DNA concentration directly after extraction; prestorage [extract] (ng/uL)—extract DNA concentration directly after extraction; poststorage [extract] (ng/uL)—extract DNA concentration after storage; [Library] (molecules)—total DNA molecules in the library (x 10^6^); seq length—library average sequence length; GC—library average GC content; total reads—total reads in the library after quality-trimming and merging; species PC1–sample loading in PC1 from the PCA based on the MetaPhlAn3 species table; species PC2–sample loading PC2 from the PCA based on the MetaPhlAn3 species table; species % PC1**+**—% of 10 species with strongest PC1 + loadings in the species-based PCA out of total species; species % PC1**−**—% of 10 species with strongest PC1-loadings in the species-based PCA out of total species; species % PC2**+**—% of 10 species with strongest PC2 + loadings in the species-based PCA out of total species; and species % PC2**−**—% of 10 species with strongest PC1-loadings in the species-based PCA out of total species.

Although we found no differences in the species profiles between heavy- and light/nonsmokers, and few associations between the species profiles and any metadata we collected from MID and CMB, we next investigated microbial gene content in the calculus from these populations. The extent to which inferred metabolic activity from ancient metagenomic data may reflect biofilm activity is an open area of investigation. Clinical periodontal microbiome research has reported distinctive gene expression profiles between dental plaque samples on teeth with and without periodontitis, even when there were not distinctive taxonomic differences (45–47). We used HUMAnN3 (48) to infer the metabolic pathways present based on gene content in the MID and CMB samples (Table S10), and performed PCA and CC analysis to assess the associations between sample metadata and the inferred metabolic potential of these calculus microbial communities (Figure 3).

Similar to the species-based CC, we found few strong correlations (> 0.4) between the PCA principal component loadings and our sample metadata (Figure 3A). The principal component loadings of PC1 were also strongly correlated with the PC1 loadings from the species-based PCA, indicating that the sample loadings are shaped by similar factors in both the taxonomic and metabolic pathway PCA. Plots of PCAs revealed no distinctive clustering of the samples based on the minimum number of pipe notches (Figure 3B), the % of teeth with periodontal disease (Figure 3C), or the library average GC content (Figure 3D). However, the samples with the highest positive PC1 loadings had the highest percentage of species with strongest positive PC1 loadings in the species PCA (Figures 2E and 3E). Finally, we investigated the species that contribute to the 10 metabolic pathways with the strongest positive and negative loadings in PC1 (Figure 3F; Table S11).

The pathways with strongest negative PC1 loadings are mainly contributed by late colonizer, anaerobic taxa in the genera *Methanobrevibacter, Desulfobulbus*, and *Desulfomicrobium* (Figure 3G). Pathways specifically attributed to archaea are here contributed nearly entirely by the archaeon *Methanobrevibacter*, and several are involved in producing cell membrane components. The pathways with the strongest positive PC1 loadings are contributed by a variety of Gram-negative aerobic and facultative species in genera including *Eikenella, Haemophilus, Kingella, Lautropia, Neisseria*, and *Ottowia* (Figure 3H), and are involved in fatty acid production (as identified in Gram-negative *Escherichia coli*), or in oxidation–reduction reactions (Table S11). The first set of pathways likely reflects the abundance of oral archaea and Gram-negative species in these samples, while the second may reflect mechanisms of protection against oxygen radical-mediated damage in an oxygen-rich environment. The difference in species contributing to pathways separating samples along PC1 reflects the gradient of taxa in the species-based PCA (Figure 2E), where samples at one end are characterized by a strong presence of early-colonizer taxa, while those at the other end are characterized by a strong presence of late-colonizer taxa. Both the species-based and metabolic pathway-based analyses indicate that calculus preserves dental plaque biofilms that calcify at different stages of biofilm development, which does not directly reflect any of the oral pathologies that we have recorded.

### Comparison with pretobacco introduction populations in Europe

While we did not detect differences in the microbial species profiles or microbial metabolic pathway profiles between heavy smokers and light/nonsmokers within the MID and CMB individuals, these populations were living during a time when smoking was common and many people would have been exposed to high levels of second-hand smoke, even if they were not using pipes or smoking themselves. High second-hand smoke exposure might obscure smoking-related species profile changes that develop between smokers and nonsmokers (49). To investigate possible differences in species profiles that are related to smoke exposure, we chose to broadly compare calculus species profiles of European populations living before and after the introduction of tobacco to Europe during three time periods: Medieval, Industrial, and present-day Modern.

We selected a total of six additional European populations based on geographic proximity and availability of comparative samples (Figure 1A). As pretobacco Medieval populations, we included the Kilteasheen calculus data set (KIL) (37) from Ireland. Additionally, we produced data from two sites in Spain dated to the medieval period, El Raval (ELR) and Iglesia de la Virgen de la Estrella (IVE), to match the CMB population. As an additional Industrial-era smoke-exposed population, we included the Radcliffe calculus data set (RAD) (4), from the early 1800s England. Modern calculus data sets from Jaen, Spain (JAE) (4) and from Valencia, Spain (VLC) (8) were also included for comparison. Poorly preserved samples were removed based on preservation assessments (Figures S2 and S3), leaving 176 samples that were used in downstream analyses (MID = 65, CMB = 8, KIL = 35, ELR = 3, IVE = 5, RAD = 42, JAE = 10, and VLC = 8).

**Fig. 4.**
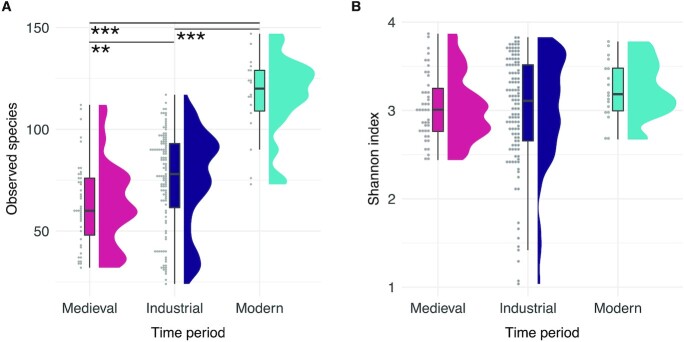
Within-sample species diversity. (A) Raincloud plots showing the observed species in each sample, grouped by time period. (B) Raincloud plots showing the Shannon index in each sample, grouped by time period. *** *P* < 0.001 and ** *P* < 0.01.

We first wanted to know if there is a change in the average number of species detected between samples from the Medieval, Industrial, and Modern periods. This was performed after removing taxa present at < 0.001% relative abundance to mitigate spurious assignments (50). Filtering affected predominantly Modern samples, as the majority of historic samples had no taxa present at < 0.001% relative abundance (Figure S12). There are statistically significant differences between Modern and Medieval groups (Figure 4, *P* < 0.001, effect size 0.71), and between Modern and Industrial groups (Figure 4, *P* < 0.001, effect size 0.48), but also between Industrial and Medieval groups, although the effect size was small (*P* < 0.01, effect size 0.24). We found few differences between historic sites, with significant differences between only MID and CMB (*P* < 0.001, effect size 0.37) and between MID and KIL (*P* < 0.001, effect size 0.41). All historic sites had significantly fewer species than either of the modern groups, VLC or JAE (Figure S7 and Table S12). The Shannon index was not significantly different between any time periods (Figure 4B) or sites (Figure S7), indicating that the distribution of species is highly similar across all samples.

To confirm the trend of increasing numbers of species in calculus samples over time, we investigated the influence of sequencing depth and average read length on species detection. The modern calculus samples were sequenced much more deeply than the historic samples, and they have a longer average read length, possibly making detection of low-abundance taxa more likely (Figure S12). We generated two additional datasets by down-sampling all calculus libraries in two ways: first by sequencing depth, then by read length. For the first set, we randomly subsampled all libraries with > 10 M reads down to 10 M reads, while maintaining all libraries with < 10 M reads (Sub10M set). For the second set, we subsampled all libraries to include only reads ≤ 75 bp in length (Sub75bp set). Each subsetted dataset was then profiled with MetaPhlAn3 and filtered to remove taxa present at < 0.001% relative abundance, and the number of species and Shannon index were calculated (Figures S8 and S9).

Both subsampling methods reduced the number of species detected for all groups (Figures S8A, S8C, S9A, and S9C), however, Modern samples still had significantly more species detected than either Medieval or Industrial samples. The Shannon index was unaffected by subsampling for read depth (Figure S8B and S8D), but was affected by subsampling for read length (Figure S9B and S9D). We found that the Sub10M dataset had significantly fewer reads than the full set for most sites (Figure S9A), but the average read length and the average GC content of the reads were unaffected (Figure S10B and S10C). The Sub75bp dataset compared to the full dataset had significantly fewer reads (Figure S10A) and significantly lower average read length (Figure S10B) for most sites, but the average GC content was minimally affected (Figure S10C). The number of species detected in the Sub10M dataset compared to the full dataset was significantly lower for both the Modern sites but none of the historic sites, while the number of species detected in the Sub75bp dataset compared to the full set was significantly lower for all sites, although two were not significantly different (Figure S11). The Modern samples in particular had fewer taxa present at < 0.001% relative abundance in the subsetted datasets than the full dataset (Figure S12). The subsampling results suggest that the higher species counts in Modern samples compared to Medieval and Industrial samples may be a real effect, but further detailed investigation of normalizing sequencing depth and read lengths, and addition of more modern calculus samples from more sites, are needed to confirm this.

**Fig. 5.**
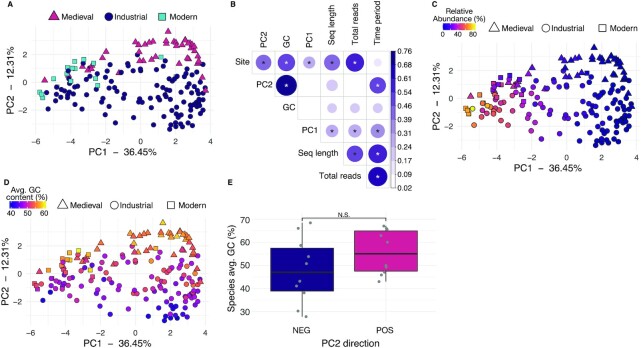
Community structure is shaped by species aerotolerance and sample GC content rather than time period. (A) PCA of species profile colored by time period. (B) CC analysis correlations between metadata categories principal component loadings for all libraries. Significance tests were performed with a Pearson correlation test. The size and color of the dots corresponds to the CC value, which does not determine the direction of the correlation (positive or negative. Hence, all CC values are positive). The tested metadata were selected because the information was available for the majority of libraries. Only correlations ≥ 0.4 have significance indicated with stars, * *P* ≤ 0.001. (C) PCA of species profile colored by relative abundance of the 10 species with strongest negative loadings in PC1, all of which are aerobic or facultative species found early in dental biofilm development (Table S2). (D) PCA of species profile colored by average GC content of the sample. (E) Average GC content of the 10 species with strongest PC1 negative (NEG) and positive (POS) loadings, indicating that species characterizing the samples with higher average GC content have higher average GC content than the species characterizing the samples with lower GC content. N.S.—nonsignificant (*P* > 0.05 by Wilcox test). Site—site of the samples; PC2–PC2 loadings; GC—library average GC content; PC1–PC1 loadings; Seq length—library average sequence length; total reads—total reads in the library after quality-trimming and merging; and time period—time period of the samples.

We next wanted to determine if the microbial community structure of calculus from Medieval, Industrial, and Modern calculus groups differ. This would let us know whether there are substantial changes that have occurred in species composition of dental calculus between distinct historic periods. We performed PCA based on the species abundance table to see whether the samples clustered by time period or by other metadata categories (Figure 5A; Figure S14A). The Medieval samples generally cluster away from the Industrial samples along PC2, while the Modern samples generally cluster with Industrial and Medieval samples at one end of PC1. While this may suggest that there are time-related differences in species composition driving separation of samples, we note several other factors that may be driving the species composition pattern and confounding this observation.

CC analysis revealed there are significant correlations between PC1 loadings and sample site, and there are significant correlations between PC2 loadings and library average GC content, in addition to site and time period (Figure 5B). We further investigated the sources of variation in the samples that most strongly influence PC loadings. Samples appear to separate along PC1 according to biofilm maturation stage (Figure 5C; Figure S14B), similar to Figure 2, however, the loadings have reversed the species gradient. The samples with more negative PC1 loadings, including all but one of the Modern calculus samples, are enriched in early colonizer, aerobic and facultative taxa such as *Streptococcus, Neisseria*, and *Rothia* (Table S4). Modern calculus samples are known to have higher levels of early-colonizer species than historic European dental calculus, which may be related to toothbrushing and other dental interventions (4). In contrast, the samples with more positive PC1 loadings are enriched in late colonizer, anaerobic, proteolytic taxa such as *Treponema, Tannerella*, and *Fretibacterium* (Figure 5C; Figure S14B and Table S4). The late-stage colonizer *Methanobrevibacter oralis* is highly abundant in several Industrial-era samples and we performed an additional PCA on a table without *M. oralis* to check if this species was strongly shaping the PCA, and found that it is not (Figure S15).

Loadings in PC2 are correlated with GC content, time period, and site (Figure 5B), which are themselves correlated. Only a single time period is represented per site, and older calculus samples are expected to have higher average GC content due to the taphonomic loss of short AT-rich fragments (37, 51). Thus it is expected that the Medieval calculus samples have higher average GC content than the Industrial samples. This is, however, not entirely an age-related effect, as the modern samples also have a higher average GC content, in the same range as the medieval samples (Figure 5D). The species characterizing samples with high positive PC2 loadings have a higher average GC content than the species characterizing samples with strong negative PC2 loadings (Figure 5E), indicating the trend in GC content is reflected in taxonomic assignments. The average read length of samples does not appear to influence the species composition of these samples, as the modern samples and RAD samples have the longest read lengths and are distributed across PC1 from the lowest to the highest values (Figure S14C). PERMANOVA testing determined clustering by site code, time period, average sequence length, and total number of reads to be significant (*P* < 0.05; Table S13), yet the R2 values for these categories are all < 0.12, indicating that the effects of these categories are too small to account for a substantial proportion of the observed variation. Taken together, it appears that the factors driving differences in species diversity between samples are derived from a variety of sources, several of which, such as GC content, are unrelated to biofilm and host ecology. These results indicate human behavioral differences such as tobacco smoking may not be as strongly reflected in calculus community profiles as they are in dental plaque, and studies will need to be carefully constructed to account for this and maximize the chance of observing signals related to the study question.

## Discussion

We investigated patterns related to dental health and pipe use in ancient dental calculus in a large osteological collection from the Netherlands, as well as a small group from Spain. No microbial species distribution patterns pertaining to dental health were detected in the samples, nor was there any indication that species profiles distinguish individuals with pipe notches (heavy smokers) and those without pipe notches (light/nonsmokers). Further, a broader time scale, spanning the medieval period through modern-day, also did not show patterns of differentiation based pre- and postintroduction of tobacco to Europe. Instead, patterns appear to be driven by individual variation in biofilm development, with individuals from each time period having species profiles ranging from more aerotolerant to more anaerobic.

Although there were some differences in dental pathologies by pipe use status within the MID collection, with users having fewer caries, more severe periodontal disease and larger calculus deposits, it was difficult to correlate the results directly with pipe smoking for two key reasons. First, the clear relationship between pipe use and biological sex, with the majority of males having at least one pipe notch and most females having no pipe notches, confounded comparison of pipe users and nonusers. In many populations, including Dutch populations of the period (52–54), there are significant differences in oral health between males and females that relate to biological differences and gendered differences in diet (55, 56). Without having more information from female pipe users and male non-pipe users it is difficult to disentangle whether the differences we see are related to biological or gender differences.

Another issue is that there is a strong correlation between age and the presence and severity of oral pathology, and because our sample was drawn from a natural cemetery assemblage, it was not balanced in terms of age: the smokers/males were older. A pertinent issue here is the inability to know whether individuals with significant tooth loss were smoking pipes. However, a previous study comparing oral pathology between MID males and earlier pretobacco males from Klaaskinderkerke identified lower caries rates, and higher AMTL and calculus severity in pipe smoking males in comparison to pretobacco males (18). Similar results were also found by others who had much larger sample sizes with a better representation of pipe users and nonusers (15, 16, 57). While these results suggest an impact, a confounding issue is that it is not possible to entirely disentangle the influence of smoking versus the abrasion of teeth by clay pipe stems, which may lead to the obliteration of early caries, hence lower caries rates, but also speed up tooth loss due to advanced wear.

The MID skeletal collection is an extensively studied assemblage, and a wealth of metadata is available for each individual. Given that dental plaque biofilm species profiles are distinct between healthy and disease-affected teeth (58), studying this collection afforded us the opportunity to assess whether ancient dental calculus microbial communities also maintain differences related to dental health. While we found no broad community-scale differences relating to pathology including periodontal disease, calculus score, caries, and AMTL, among others, this is consistent with the observation that dental calculus appears to be a stable climax biofilm community (4). The ecological succession by which biofilms reach a terminal state (59–65) may be influenced by external forces including oral hygiene, immunological responses, and tobacco use, with intermediate biofilm states strongly depending on the local oral environment (38). However, once a terminal community is reached, the fully mature biofilm may have lost most indications of earlier community states.

Tobacco smoking adversely affects oral health, and is associated with substantial changes in dental plaque biofilm community profiles (66, 67). Whether the microbial changes are due to direct influence of tobacco smoke exposure or to physiological changes in the oral tissues is not clear, and different studies report different specific species abundance changes (32–36). Overall, however, the species profile appears to shift to one with more pathogenic potential, with elevated abundances of anaerobic, proteolytic species that are associated with periodontal disease. Given that dental calculus species profiles generally have higher abundance of these same disease-associated species, even in the absence of dental pathology (4, 68), detecting smoke exposure-induced changes may be difficult. Specific changes might be detected by differential abundance analysis, but the relative value of this information in ancient dental calculus remains to be investigated.

The patterns we detected within the MID and CMB samples appear to be related to individual differences in the terminal dental biofilm community. The results of our transect support this observation, which is consistent with other publications that have performed time transect studies (8, 10), where community diversity analyses do not cluster samples by time period. Instead of patterns relating to sample site, time period, or dental health, the samples appear to maintain individuality. This pattern of stable microbial signatures in dental plaque is a known phenomenon, with individual teeth having relatively stable communities over months-long time scales (69). Despite high variability in profiles across teeth within an individual, plaque from any one tooth in an individual is more similar to other teeth in the same individual than to teeth in other individuals (69). This pattern of individuality is also captured in ancient dental calculus (44), where there is relatively high variability across teeth in an individual, yet samples from one individual are more similar to each other than to samples from another individual.

The strongest pattern we detected separating samples in beta-diversity analysis was the proportion of species with different environmental niches. This was shown by a strong gradient where samples at one end of the PCA were enriched in aerotolerant, early biofilm colonizer taxa, and samples at the opposite end were enriched in anaerobic, later biofilm colonizer taxa. This was true for not only the MID and CMB samples, but also for samples from other sites and time periods in Europe. We found no correlation with dental pathology, suggesting individual differences in plaque development timelines may account for this pattern, and studies of biofilm succession may offer insight. In vivo studies of plaque development have reported two distinct patterns, one termed “rapid” and the other “slow” (60, 70, 71). Slow plaque-forming individuals maintained a “younger” biofilm dominated by more aerotolerant taxa, including *Streptococcus* and other Gram-positive cocci (60, 70, 71). During the same period of time, the biofilm in rapid plaque formers developed a more complex community with higher proportions of anaerobic and Gram-negative taxa. This difference in the rate at which biofilms mature and calcify, and the species composition of the end-state climax community, may be captured in dental calculus.

The results of this study suggest that it may not be possible to use dental calculus metagenomes to distinguish broad health-associated changes in community profiles such as can be detected in living dental plaque biofilms. Instead, the value of these ancient metagenomes may lie in providing insight into individual biofilm characteristics, particularly related to biofilm and microbial ecology. Another intriguing avenue of ancient dental calculus metagenome research is on the evolution of specific species and strains through the assembly and reconstruction of ancient metagenome assembled genomes (9, 72, 73). In contrast, the wealth of proteins and metabolites that are preserved in calculus may reflect biofilm community responses to altered oral environments such as dental disease or tobacco smoking (3, 4, 74), and could be used instead of the metagenome to study the role of health in shaping the oral microbiome in deep time.

## Materials and Methods

### Archaeological sites and associated skeletal remains

#### MID

Total DNA from the calculus of 75 Beemster individuals was extracted and sequenced for this study. The oral health of each skeleton was recorded with reference to the presence and absence of pipe notches, periapical lesions, supra- and subgingival calculus scores, periodontal diseases, caries, AMTL, and their respective severeness (Table S1). To be scored in the analysis of dental pathology, it was important to know whether an individual had pipe notches or not. As such, only individuals retaining at least 50% of their anterior teeth in alternating positions (thereby allowing pipe notches to be observed) were included in the analysis of dental pathology. To evaluate the presence and severity of periodontal disease, the mandible and maxilla of each individual were examined macroscopically. Changes were recorded based on the inflammatory loss of the alveolar bone, and scored following the four point scale described in (75): none–1, mild–2, moderate–3, and severe–4. Additionally, the percentage of tooth positions with signs of periodontitis was calculated. For details of metadata collection see “Supplemental Methods.”

Dental calculus from the MID collection was collected by K.Z. in 2014. For each individual, calculus from multiple teeth was sampled and pooled for analysis. Calculus from a total of 75 individuals was sampled. Of these, 40 individuals had pipe notches, and seven individuals had dentitions that were too fragmentary to determine whether pipe notches were present.

#### CMB

Calculus from eight individuals from the CMB cemetery were extracted and sequenced for this study. A total of four individuals had pipe notches in their dentition, and four did not. It is possible that individuals CMB003 and CMB004 are the same individual, as each is represented by a partial mandible recovered from the same grave. The oral health of each skeleton was recorded with reference to the presence and absence of pipe notches. This was investigated as previously described (76). For details see “Supplemental Methods.” Dental calculus from the CMB was collected by M. I. G-C. in 2019. For each individual, calculus from multiple teeth was sampled and pooled for analysis. A total of eight individuals were sampled, four with pipe notches, and four without pipe notches (Figure S1).

ELR and IVE. Dental calculus was also analyzed for two Spanish sites dated to the Medieval period, ELR and IVE. Calculus from five individuals from each site was extracted and sequenced for this study (Table S1). Samples from ELR were collected in 2018 by D.C.S.G. Calculus were collected off of multiple teeth and pooled for extraction/collected off a single tooth per individual (77). Samples from IVE were collected in 2016 by D.C.S.G. Calculus were collected from multiple teeth and pooled for extraction.

Metadata for the samples sequenced for this study are in Table S1.

### Comparative data sets

A total of two published datasets were selected to represent comparative European populations pre- and postintroduction of tobacco to Europe: the KIL dataset from Ireland ca. 600 to 1,300 CE (*n* = 36, (37)), and the RAD Infirmary burial ground set from England ca. 1,850 (*n* = 44, (4)). Data from Chalcolithic-era dental calculus (ca. 4,500 to 5,000 BP) previously reported in Fagernäs et al. (44) were included as a comparative dataset to examine intraindividual variation in *Streptococcus* species. The samples are listed in Table S5.

### DNA extraction

For each MID dental calculus sample, 10 to 30 mg were subsampled for extraction into 1.5 ml tubes. To remove surface contamination, 1 ml of 0.5 M EDTA was added to each sample and mixed by vortexing for 20 seconds, followed by 15 minutes of incubation with rotation. The sample solutions were then briefly centrifuged for 1 minute at 6,000 rpm (batches 1 to 4) or 13,000 rpm (batch 5) to pellet the calculus fragments, and the supernatant was removed. Samples were extracted using a previously published phenol: chloroform etraction method (78). For details see “Supplemental Methods.”

DNA extractions from the CMB, ELR, and IVE sites were performed following the published protocol “Ancient DNA Extraction from Dental Calculus” (79). A single extraction blank, which included water instead of a sample, was included in each extraction batch.

### Library building and sequencing

Library preparation, indexing, amplification, and pooling for all extraction sets (MID, CMB, ELR, and IVE) was identical. Library preparation was performed following the published protocol “Half-UDG treated double-stranded ancient DNA library preparation for Illumina sequencing” (80), including a single library blank per batch, which included water instead of sample extract. Indexing was performed following the published protocol “Illumina double-stranded DNA dual indexing for ancient DNA V.2” (81), modified from (82). Final amplification and pooling were performed following the published protocol “Amplification and Pooling” (83). Sequencing of the pooled libraries was performed on two flow cells on an Illumina NextSeq500, with 2 × 75 bp chemistry to a depth of ∼8 M reads per calculus library and ∼2 M reads per blank library (Table S1). For additional details see “Supplemental Methods.”

### Data processing

All raw data were processed using the nf-core/eager pipeline (84), version 2.1.0. This included quality checks with FastQC (85), adapter trimming, read collapsing, and quality filtering with AdapterRemoval (86), and mapping against the human genome (HG19) with bwa aln -n 0.02 -l 1024 (87) and samtools (88) to remove human reads. The human-mapped reads were not used for any analyses. Taxonomic profiling was performed with MALT v. 0.4.0 (89, 90). Additional details are in the “Supplemental Methods.” Metadata for data processing for data produced for this study are in Table S6.

### Processing of comparative data

Published historic dental calculus samples were downloaded from ENA and processed with the nf-core/eager pipeline (84), described above. We included the KIL calculus data set (37) from medieval Ireland, the RAD calculus data set (4), from the early 1800s England, modern calculus data sets from Spain (JAE and VLC) (4, 8), and the four Chalcolithic individuals from (44). Metadata for data processing for published data used in this study are in Table S7.

### Taxonomic profiling and decontamination

All remaining reads that did not map to the human genome were taxonomically profiled with two profilers: MetaPhlAn3 (48) run as a stand-alone program, and MALT v. 0.4.0 (89, 90) within the nf-core/eager pipeline. All microbial species diversity and correlation analyses, as well as cuperdec (8) preservation analysis, were performed with the MetaPhlAn3 species table. Analysis with the MALT table produced a distinctive horseshoe-shaped PCA (Figure S5), indicative of a taxonomic gradient in our samples (91), which made interpretation of associations with metadata difficult. Therefore, the MALT table was not used for taxonomic analysis, and instead the MetaPhlAn3 table was used. Sample preservation was assessed using cuperdec (8), decontam (92), and SourceTracker (93). For details see “Supplemental Methods.”

### Diversity analyses and metadata comparisons

The MetaPhlAn3 species table was used for all diversity analyses. For alpha-diversity analyses, the tables were filtered to include only species present at > 0.001% abundance. This affected mainly the modern JAE and VLC samples, while the majority of historic samples had no taxa present at < 0.001% abundance (Figure S12). The Shannon index was calculated in R using the diversity function in the package vegan (94). Kruskal–Wallis tests were performed using the R package rstatix (95). A PCA was calculated in R on an unfiltered CLR-transformed species table using the package mixOmics (96). PERMANOVA was run on the PCA using the function adonis2 in the R package vegan (94). Filtering was found to have almost no effect on the proportion of variance explained in PC1 and PC2 of the PCAs, and PERMANOVAs performed on PCAs of filtered tables produced nearly identical R2, F, and *P*-values (Figure S13, Tables S9 and S13). Batch effects within the MID sample set were investigated by coloring the points in a PCA plot by extraction batch, but no clustering based on batch was observed (Figure S4).

### Subsampled datasets

The effects of library sequencing depth and average read length on the number of species detected were investigated by down-sampling the full libraries in two ways: (1) randomly subsampling all libraries with > 10 M reads down to 10 M reads, while leaving all libraries with < 10 M reads untouched (Sub10M set), and (2) subsampling all libraries to include only reads ≤ 75 bp in length (Sub75bp set). Alpha-diversity metrics Observed species (number of species) and Shannon index were calculated as described for the full set in Diversity analyses and metadata comparisons (Table S12). For additional details see “Supplemental Methods.”

### CC analysis

CC analysis was performed to look for correlations between different metadata categories and between metadata and principal component loadings from PCA, as in (97). Input tables contained selected metadata categories and the PC1 and PC2 loadings from PCA. CC were calculated with the function canCorPairs from the R package variancePartition (98, 99). Statistical tests were performed with the cor.mtest function in the R package corrplot (100), and correlation matrix plots were generated with the function corrplot in the same package. To focus on the strongest correlations, we considered only correlations ≥ 0.4 with a significance of *P* ≤ 0.05.

### HUMAnN3 functional analysis

Potential metabolic functional profiles were generated with HUMAnN3 (48) using default parameters. We used the pathway abundance table, which was converted from reads per kilobase to copies per million with the humann3 helper script humann_renorm_table.py (Table S10). The total pathway assignment per sample was used in analysis, and not the species-specific assignments per pathway. A PCA and CC analysis were performed on the pathway abundance table as described for the species tables above.

### Additional plotting aspects

Plots were arranged in grids using cowplot (101) or patchwork v1.1.0 (102) in R. Metadata plots used ggpointgrid (103). Significance on plots was indicated with the R package ggsignif (104). A map of Europe with the sites of MID, CMB, and comparative data sites was generated in R using the packagtes sf (105), rnaturalearth (106), rnaturalearthdata (107), rgeos (108), and maps (109). Scripts to generate all main and supplemental figures can be found in their respective folders in the github repository: https://github.com/ivelsko/smoking_calculus/.

## Supplementary Material

pgac148_Supplemental_Files

## Data Availability

All data generated for this study has been uploaded to the European Nucleotide Archive under accession PRJEB52394. Scripts for analysis can be found on the github repository https://github.com/ivelsko/smoking_calculus.
