# Supplementary material for: Ancient dental calculus preserves signatures of biofilm succession and interindividual variation independent of dental pathology
Source: PNAS Nexus. 2022 Aug 4;1(4):pgac148. doi: 10.1093/pnasnexus/pgac148 (PMC9802386; doi:10.1093/pnasnexus/pgac148)
Supplement: pgac148_Supplemental_Files [file pgac148_supplemental_files.zip › PNASNEXUS-PNASNEXUS-2022-00453-s01.pdf]

Supporting Information for

Ancient dental calculus preserves signatures of biofilm succession and inter-individual variation independent of dental pathology

Irina M. Velsko, Lena Semerau, Sarah A. Inskip, Maite Iris García-Collado, Kirsten Ziesemer, Maria Serrano Ruber, Luis Benítez de Lugo Enrich, Jesús Manuel Molero García, David Gallego Valle, Ana Cristina Peña Ruiz, Domingo Salazar Garcia, Menno L.P. Hoogland, Christina Warinner

Supplemental Methods 3

List of Supplemental Tables in Excel sheets 8

Table S2. Number of pipe notches in individuals from Middenbeemster. 9

Table S3. Numbers of observations and prevalence rates for oral pathologies for males, females, and a pooled sex group. 10

Figure S1. Pipe notches in anterior maxillary dentition of 2 individuals from Convento de los Mercedarios de Burtzeña (CMB). 11

Figure S2. Preservation assessment. 12

Figure S3. SourceTracker plot showing in each sample the proportion of species assigned to each source. 13

Figure S4. PCA plot of species diversity for Middenbeemster (MID) and Convento de los Mercedarios de Burtzeña (CMB). 14

Figure S5. Species gradient in Middenbeemster (MID) and Convento de los Mercedarios de Burtzeña (CMB) calculus samples profiled with MALT. 15

Figure S6. PCA of Middenbeemster and Convento de los Mercedarios de Burtzeña samples, colored by the location of teeth off of which calculus was collected. 16

Figure S7. Alpha-diversity of samples grouped by site. 17

Figure S8. Alpha-diversity after sub-sampling all libraries to a maximum of 10M reads. 18

Figure S9. Alpha-diversity after sub-sampling all libraries for only reads 75 bp. 19

Figure S10. Read characteristics for full and sub-sampled libraries. 20

Figure S11. Diversity indices for full and sub-sampled libraries. 21

Figure S12. Species removed per sample by filtering the MetaPhlAn3 species tables to remove all species present at < 0.001% abundance. 22

Figure S13. PCAs based on unfiltered MetaPhlAn3 data tables. 23

Figure S14. Beta-diversity PCA plot of calculus samples from pre- and post-introduction of tobacco to Europe. 24

Table S4. Top 10 species with strongest loadings in PC1 in the PCA with pre- and post-tobacco introduction to Europe samples. 25

Figure S15. PCA of the species table excluding *Methanobrevibacter oralis*. 26

References **27**

Supplemental Methods

Archaeological sites

Middenbeemster (MID). Middenbeemster is today a small town in the Beemster region of the Dutch province of North Holland (Figure 1A). Settlement in the region began after the draining of the Beemster lake in 1612 (Aten et al. 2012). Following land reclamation, the region was used primarily for agriculture, and it became particularly renowned for its dairy farming and cheese production (1), although residents also worked in local service industries, shops, and the military (2). While it was initially planned to construct multiple churches throughout the region, only one church, the Keyserkerk, was completed at Middenbeemster, and as such it served the entire Beemster community (3).

In 2011, the Laboratory for Human Osteoarchaeology of Leiden University and the company Hollandia Archeologen carried out archaeological excavations at the Middenbeemster Keyserkerk prior to a planned construction project. The excavation of part of the cemetery (4), which was in use from 1623 to 1866, uncovered approximately 450 individuals, and their skeletal remains were relocated to the Human Osteology Laboratory of Leiden University for research and long-term care. Most of the excavated individuals date to the later periods of the cemetery's use because historical records indicate that the older portions of the cemetery, which had become overcrowded, were cleared in 1829 to make room for new burials (4). At the time of death, undertakers and gravediggers wrote down individual information for each burial, including name, age at death, date of death, and the profession of the deceased, creating burial records, death registers, and a map of Middenbeemster's cemetery (5). This information was used to identify some of the excavated skeletons; however, alternative ways of spelling names and typographical errors have made it difficult to identify most remains with certainty (4). After excavation, the skeletal remains recovered from the cemetery were washed with water to remove clay and sand from the burial prior to storage.

All metadata were collected by the Laboratory for Human Osteoarchaeology of Leiden University and confirmed by S. I. and M. H.. Biological sex estimates were made by observing and recording the sexually dimorphic morphological traits of the skull and pelvis, following standard methods (6, 7). The approximate age at death for each individual was estimated using dental attrition (8), cranial suture closure (9), the sternal rib-end (10), the pubic symphysis (11), and the auricular surface (12) and grouped into four age categories: adolescent (13-18 years), young adult (18-25 years), middle adult (25-45 years), and old adult (>45 years). For known individuals, age estimates were cross checked with archival information, which showed a high degree of accuracy for sex estimates, and good accuracy with age estimates.

Convento de los Mercedarios de Burtzeña (CMB). The Convento de los Mercedarios de Burtzeña, is located in the eastern part of Burtzeña, in Northern Spain. Archaeological and historical information about the site and its excavation are available in (13) and (14). The use of the cemetery is dated between the end of the 16th century. and the beginning of the 19th

century. The excavations revealed the remains of the foundation under the pavement of the church, which contained the sandstone-boxed inhumations of more than 150 graves, of which most were occupied by several individuals. Fifty graves, covered with slabs, were excavated during the excavation works. A total of 62 articulated individuals and numerous scattered bone remains were found. Each grave contained individuals of different ages, which may represent family graves, which were typical at that time.

All metadata for the site were investigated and collected by M. I. G-C., E. D. B. and L. S. Z.. All the anthropological remains recovered were cleaned by washing with water at room temperature and brushing with soft bristled brushes. Care was taken not to submerge the bones completely in order to minimize water absorption. The material was then left to dry at room temperature on absorbent paper in a ventilated room.

Biological sex was estimated observing the sexially dimorphic morphological traits of the skull and pelvis (6, 15). Age-at-death of each individual was estimated using the pubic symphysis (11), the auricular surface (9), the sternal rib-end (10) and the sacrum (16). Since most individuals originated from disarticulated assemblages of human remains and often were just isolated mandibles, it was not possible to make precise age estimations for all skeletons. Four individuals could only be classified as “older than 20 years” and therefore could not be classified into the same age groups as used for the Middenbeemster collection; they were instead classified as "Adult".

El Raval. El Raval is a medieval necropolis from the city of Crevillent (Alacant, Spain), from the times of the Kingdom of València, Crown of Aragón. The necropolis was radiocarbon dated between the end of the 14th century AD and the beginning of the 16th century A.D., and was located outside the city wall besides one of the main roads leading into the city (17). Burial customs revealed that most individuals interred were Mudéjar, Muslims of Al-Andalus that remained in Iberia after the Christian Conquest, and a minority were Islamic people who had converted to Christianity (18). A total of 81 burials were recovered, mostly single graves occasionally covered by rocks or wood. Individuals of all ages, except those older than 60, were buried in the cemetery, being both full adults and infants (0-4 yo) the most frequent. Oral pathologies, such as dental calculus, caries, periodontal disease and antemortem tooth loss, are frequent amongst the adult individuals (19).

Tobacco use at the sites

European encounters with tobacco commenced in the15th and 16th century during the European colonization of the Americas, at which time it was presented to them by indigenous American peoples (20, 21). Tobacco was long used by indigenous peoples in a myriad of ways; it formed an important part of their lives and identities. Its use as a medicinal agent, inspired Europeans to investigate its healing and curative properties, while its role in ritual and political ceremonies demonstrated its use as a social and recreational entity (21). In terms of smoking, the habit traversed the Atlantic via colonialists, returning adventurers and sailors, sojourners, Indigenous delegates, and enslaved individuals. From there it diffused into the general population (21–23). By the early 17th century, tobacco was a taxable commodity thorughout much of western Europe

including present-day Spain, England, France, and the Netherlands, and was consumed by a large proportion of society (23).

Tobacco smoking is well documented in the Netherlands during the 17th to 19th century. Initially, the Dutch procured much of their tobacco from the English, although later they had their own domestic industry (22). While there were changing fashions, clay pipe smoking was the dominant method for tobacco use in the Netherlands, who became the leading producers of pipes in Europe (24). Pipe smoking was a common habit that was associated with masculine identity and sociability, together with the consumption of alcohol (Brongers 1964). During excavations of the cemetery at Middenbeemster, eleven 17th-19th century clay pipe fragments were recovered from a ditch in the cemetery boundaries (25). There are also multiple contemporaneous sites that have been excavated in the Beemster that have yielded abundant clay pipe fragments (26). Furthermore, advertisements indicate that there was also a cigar manufacturing company in the Beemster, making cigars also available to the local population (27). Snuff and tobacco were also used as ingredients in medical remedies.

Tobacco was economically significant in the post-medieval period and as a result there are plentiful historical sources on its import, processing and use. The kingdoms of Castille and Portugal had the earliest colonies and were exporting tobacco to Europe by the end of the 16th century (21). In the early 17th century, all tobacco imports came through Seville (23) which also became the leading producer of European snuff. In contrast to the Netherlands, the inhabitants of Iberia were renowned for their cigars and snuff, which likely relates to their early encounters with indigenous peoples who used tobacco in this form (23). However, depictions of tobacco pipe smoking (21) and the finding of tobacco pipes at Post-Medieval and Modern sites showing that people used it in this form (28, 29). The graves at the CMB site were found to contain kaolin clay pipes, in addition to coins, rosaries, crosses, medals, remains of ceramics, glass, metal, nails, and fragments of stained glass. Among the many clay pipes recovered, one fragment contained a type of decoration suggesting that it may have originated from a pipe of Dutch manufacture. These clay pipes became popular in the region in the 17th century (13).

DNA extraction

<u>Middenbeemster</u>

To decalcify the decontaminated dental calculus, the resulting pellet was resuspended in 1 ml of 0.5 m EDTA and vortexed for 20 seconds. All samples were then incubated with rotation for 4.75 hours (batch 5), 7 hours (batch 2) or overnight (batches 1, 3, 4). To each sample, 100 µl Proteinase K (30 units/mg) was added, while the controls received 50 µl. All tubes were incubated at 55°C for 5.5 hours (batch 1), 6.5 hours (batch 4), 7.5 hours (batch 3) or overnight (batches 2, 5). There was only enough Qiagen Proteinase K to add to Batch 1-3, therefore Batch 4 and 5 needed to be treated with Invitrogen Proteinase K that has been dissolved in 2.5 ml 99.5% glycerol, 0.5 ml Tris-HCl (100 mM), 0.1 ml CaCl (1M) and 1.9 ml H2O. The samples were held at room temperature and further incubated and digested for five days.

The extraction of the MID DNA was performed using the phenol:chloroform:isoamyl alcohol (25:24:1) method. Solutions B1 and B2 consisted of 375 µl phenol and 375 µl chloroform:isoamylalcohol (phenol:chloroform:isoamlyalcohol 25:24:1), B3 of 750 chloroform: isoamylalcohol. The samples were centrifuged at 13000 rpm for 5 minutes. The supernatant was transferred to solution B1 and the pellet was stored at -20°C. The B1 mix was incubated while being rotated for 1 minute. After a centrifugation step at 13000 rpm for 5 minutes, the aqueous phase was transferred to solution B2, and the rotation and centrifugation step was repeated. The organic phase of B1 was stored at - 20°C. Again, the aqueous phase of B2 was transferred to B3, the mixture rotated and centrifuged and the organic phase of B2 stored at - 20°C.

The extracted DNA was isolated by silica column-based purification. A MinElute Zymo reservoir with 13 ml PB buffer was placed in a 50 ml falcon tube. The sample was transferred to the PB buffer in the column and centrifuged at 1500 g for 4 minutes, then rotated for 2 minutes. The MinElute column was removed from the reservoir and transferred in a clean collection tube. The column was dry spun at 6000 rpm for 1 minute, and the flow-through was discarded. The DNA containing membranes were washed twice by adding 750 µl PE buffer and centrifugation at 6000 rpm for 1 minute. Each time, the flow-through was discarded. The column underwent another dry spin at 13000 rpm for 1 minute. The MinElute column was transferred to a clean collection tube. A volume of 30 µl EB buffer was added to the center of the filter of the column and incubated for 5 minutes. To elute the DNA from the column, the columns were centrifuged at 13000 rpm for 1 minute. The flow-through was collected, quantified using a Qubit fluorometer and stored at -20°C.

Taxonomic profiling
The complete MetaPhlAn3 table can be found in Supplemental table S8. The commands to generate the metaphlan3 table can be found in /mnt/archgen/microbiome_calculus/smoking_calculus/02-scripts.backup/009-metaphlan3_mid.Snakefile. MALT was run within the nf-core/eager pipeline with default settings. The database used was the custom RefSeq database described in Fellows Yates (30). The output rma6 files from MALT were imported to MEGAN6 CE v. 6.18.0 (31) with the "comparison" mode, and a species-level table with read counts was exported as a tsv file for all downstream analyses.

Sample preservation analysis
Poorly preserved dental calculus samples were identified using the R package cuperdec (30) using the MetaPhlAn3 table, and were removed from the table for all downstream processing. Potential contaminant taxa were removed from the taxonomic table using the R package decontam (32), with extraction blanks and library blanks from this study, as well as femur samples from (30), as controls. Scripts for these steps can be found in the github repository: https://github.com/ivelsko/smoking_calculus/02-scripts.backup/ in the files MID_mpa3_cuperdec.Rmd, MID_mpa3_decontam.Rmd.

SourceTracker (33) was used to determine the proportion of species in each sample that come from a set of authentic oral and potential contamination sources. SourceTracker was run using the MALT species table as input because species profiles of the source sample generated with MALT using the RefSeq database from (30) were available, negating the need to download, process, and taxonomically profile the source raw data. Species tables for all sources except modern calculus were obtained from the table Evolution-Comparison_MEGAN_20190410-ex_absolute_species_prokaryotes_summarised_refseq.txt from the github page of Fellows Yates, et al. (30), and the following sources were used: modern dental calculus, supragingival plaque, subgingival plaque, rural gut, urban gut, skin, archaeological bone, and sediment. Modern calculus source data was obtained from Velsko, et al. (34) and Fellows Yates, et al. (30) and was processed through nf-core/eager and MALT with the ancient calculus for this study. Scripts for these steps can be found in the github repository: https://github.com/ivelsko/smoking_calculus/02-scripts.backup/ in the files 001-shotgun_sourcetracker_high_rare.sh and MID_tax_sourcetracker.Rmd.

Subsampled datasets

The effects of library sequencing depth and average read length on the number of species detected were investigated by down-sampling the full libraries in two ways. For the first set, we randomly subsampled all libraries with > 10M reads down to 10M reads using seqtk and setting the seed to -s10000, while leaving all libraries with < 10M reads untouched (Sub 10M set). For the second set, we subsampled all libraries to include only reads ≤ 75bp in length (Sub 75bp set) using bioawk -c fastx '{if (length($seq) < 76){print "@"$name" "$comment"\n"$seq"\n+\n"$qual}}' <library>. The full libraries that were downsampled were those that had been processed by nf-core/eager (adapter-trimmed and quality-filtered, collapsed, mapped against the human genome, and had human reads removed), and were profiled by MetaPhlAn3 for full analysis. Both subsetted datasets were profiled with MetaPhlAn3 as described above for the full set. The total number of reads, the average read length, and the average GC content of the libraries for both subsetted datasets were calculated from FASTQC (Andrews, 2010) using multiqc (Ewels et al., 2016) (Supplemental Tables S6, S7) and compared with the full set.

List of Supplemental Tables in Excel sheets

Table S1. Oral pathology metadata for samples sequenced for this study. (Excel sheet)

Table S5. Oral pathology metadata for published samples included in this study. (Excel sheet)

Table S6. Extraction and library metadata for samples sequenced for this study (Excel sheet)

Table S7. Extraction and library metadata for published samples included in this study (Excel sheet)

Table S8. MetaPhlAn3 taxonomy table for all samples included in this study. (Excel sheet)

Table S9. PERMANOVA test results for Middenbeemster sample dataset profiled with MetaPhlAn3 and metadata (Excel sheet)

Table S10. HUMAnN3 pathway abundance table for all samples included in this study. (Excel sheet)

Table S11. HUMAnN3 pathways with strongest loadings in PC1 and PC2. (Excel sheet)

Table S12. Alpha-diversity values for all samples profiled with MetaPhlAn3. (Excel sheet)

Table S13. PERMANOVA test results for all samples dataset profiled with MetaPhlAn3 and metadata (Excel sheet)

Table S2. Number of pipe notches in individuals from Middenbeemster.

		Number of pipe notches							
		**0**	**1**	**2**	**3**	**4**	**5**	**7**	**Total**
Biological sex	Female	22	2	1	-	-	-	-	25
	Indeterminate	2	-	2	-	-	1	-	5
	Male	5	9	10	4	7	3	1	39
	Total	29	11	13	4	7	4	1	69

(-) indicates no individuals fell into this category.

Table S3. Numbers of observations and prevalence rates for oral pathologies for males, females, and a pooled sex group.

	Males			Females			Pooled sex		
	All Male	Pipe notches absent	Pipe notches present	All female	Pipe notches absent	Pipe notches present	All	Pipe notches absent	Pipe notches present
**Number of individuals**	40	4	36	25	22	3	70	28	42
**AMTL**									
Number observable for AMTL	39	4	35	25	22	3	69	28	41
% individuals with AMTL	82	75	82.8	64	68.2	33.3	75.4	67.9	80.5
%teeth lost AMTL	13.3	14.2	13.2	12.6	13.2	8.3	13	12.6	13.3
**Periapical lesions**									
No ob periapical lesions	39	4	35	25	22	3	68	28	40
% individuals with periapical lesion	51.3	50	51.4	32	36.4	0	41.1	35.7	45
Average of No positions with periapical lesion	3.8	2.3	3.9	2.8	3.2	0	3.2	2.8	3.4
**Caries**									
No individuals observable for caries	38	4	34	25	22	3	67	28	39
%individual with caries	79	100	76.5	88	86.3	100	82	85.7	79.5
%individual Gross caries	31.6	50	29.4	52	54.6	33.3	36.8	50	27.5
Average of %teeth with caries	14.9	15.1	12.9	28.7	30.5	15.4	19.6	26.03	15.1
**Calculus**									
No individuals observable for calculus	38	4	34	24	21	3	66	27	39
% individuals with calculus	100	100	100	100	100	100	100	100	100
% of teeth with calculus	69.3	42.7	72.4	63.3	62.7	67.6	67.3	58.6	73.5
Average of calc sup score	2.4	1.5	2.5	2.2	2.1	3	2.3	2	2.5
Average of Calc sub score	2.2	1.75	2.3	1.8	1.8	1.7	2	1.8	2.2
**Periodontal disease (PD)**									
No individuals observable for PD	39	4	35	25	22	3	68	28	40
% individuals with PD (score 3 or 4)	87.5	75	89	64	64.6	66.7	78.3	64.3	87.8
Average max PD score	3.2	3	3.2	2.9	2.9	3	3.1	2.9	3.2
Average of % positions with PD	74.3	69.8	74.9	62.4	66.1	37.8	69	64.3	72.2

No - number; **AMTL** - antemortem tooth loss; **sup** - supragingival; **sub** - subgingival; **PD** - periodontal disease.

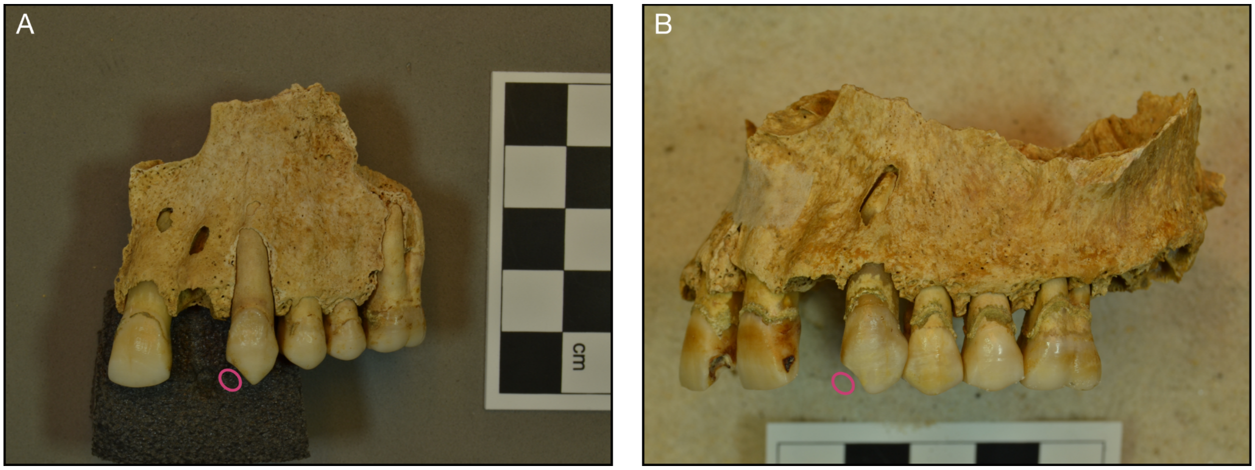

Figure S1. Pipe notches in anterior maxillary dentition of 2 individuals from Convento de los Mercedarios de Burtzeña (CMB). **A.** Individual CMB001. **B.** Individual CMB003. Notch locations are indicated by hollow pink circles. Photo credit: Maite I. Garcia-Collado.

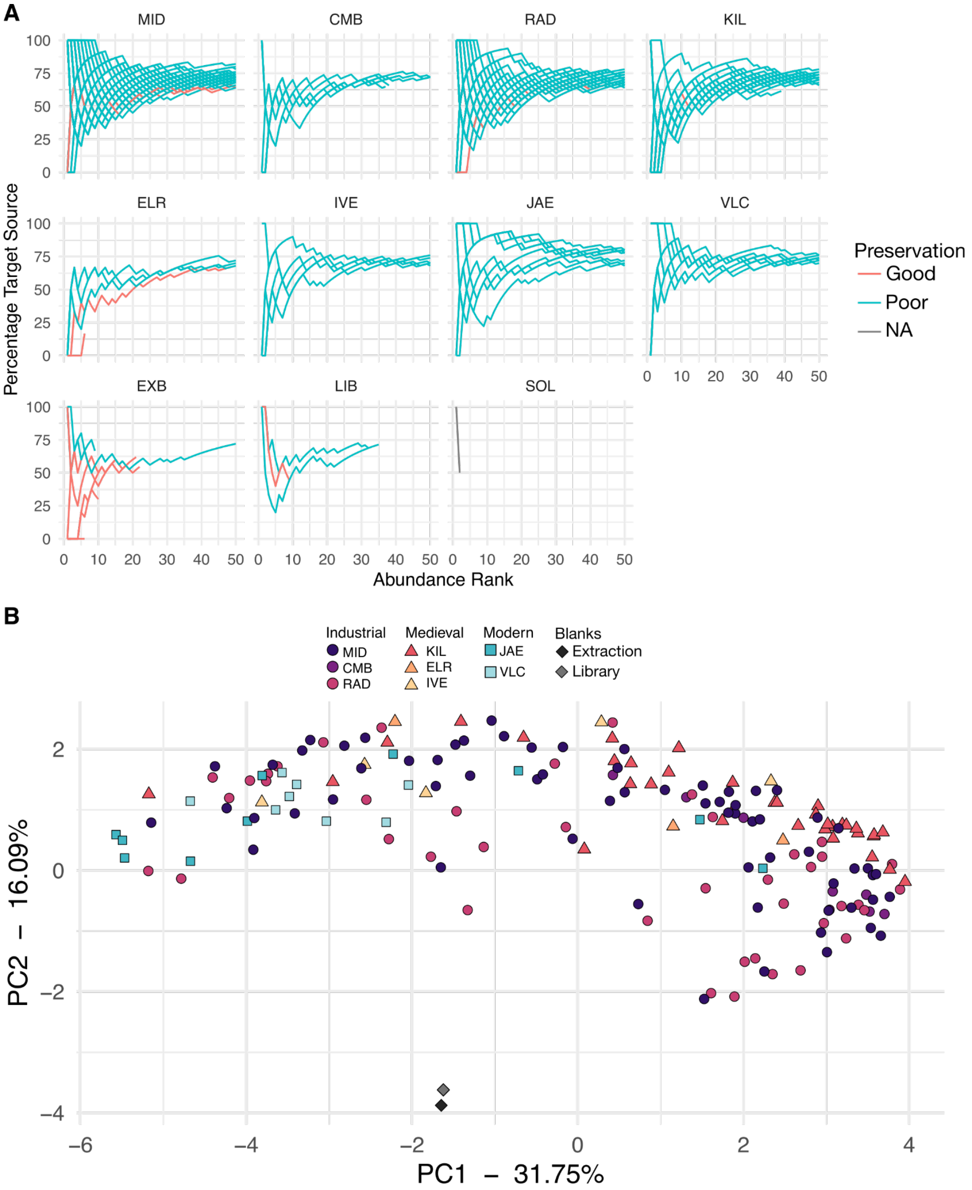

Figure S2. Preservation assessment. A. Cumulative percent decay curves (cuperdec). Curves demonstrating the proportion of species that come from an oral source, ordered by abundance. All samples with red lines were removed from all analyses. B. PCA of samples passing cuperdec preservation cut-offs, as well as extraction and library blanks. Site codes: **MID** - Middenbeemster, **CMB** - Convento de los Mercedarios de Burtzeña, **RAD** - Radcliffe, KIL - Kilteasheen, **ELR** - El Raval, **IVE** - Iglesia de la Virgen de la Estrella, **JAE** - Jaen, **VLC** - Valencia, **EXB** - Extraction blank, **LIB** - library blank, **SOL** - soil.

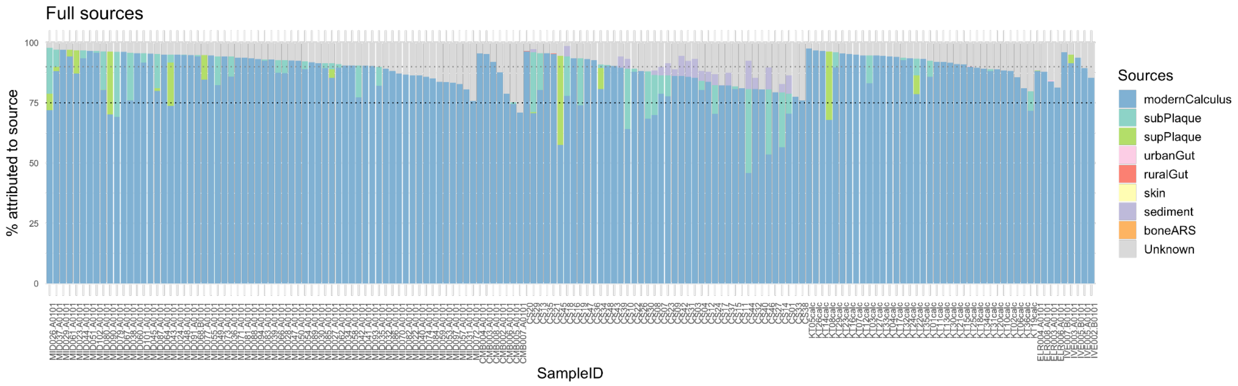

Figure S3. SourceTracker plot showing in each sample the proportion of species assigned to each source. Only a single sample has a proportion below 75% assigned to an oral source (modern calculus, subgingival plaque, supragingival plaque). The input table was from a MALT run that used the RefSeq database from Fellows Yates, et al. 2021. SubPlaque - subgingival plaque, supPlaque - supragingival plaque, boneARS - bones from site Arbulag sum, Mongolia (site code ARS). The modern calculus used as a source is the JAE samples used in this study as comparative samples. The dotted black line indicates 75%, and the dotted gray line indicates 90%.

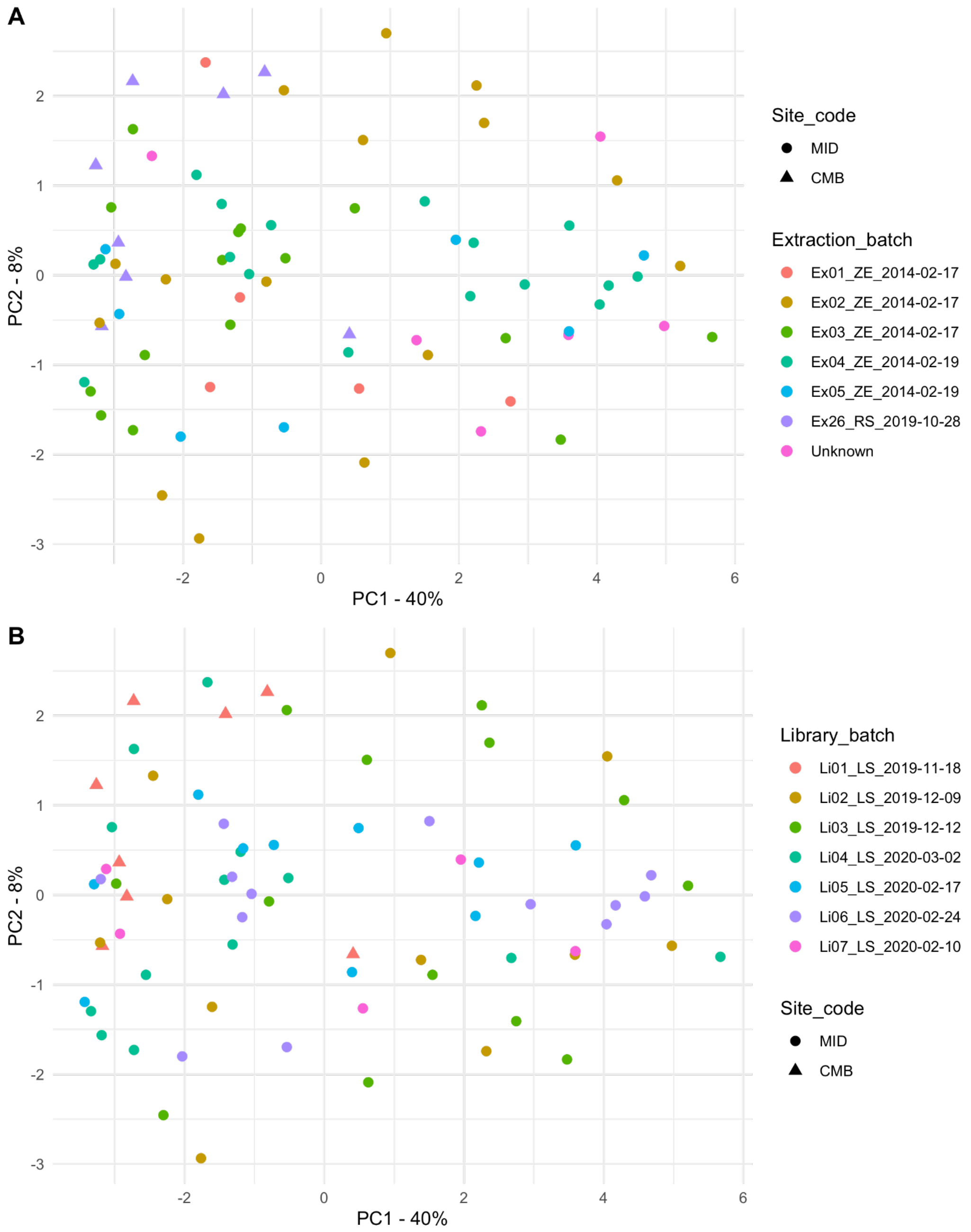

Figure S4. PCA plot of species diversity for Middenbeemster (MID) and Convento de los Mercedarios de Burtzeña (CMB) colored by **A.** extraction batch and **B.** library batch. Samples do not cluster by either extraction or library batch.

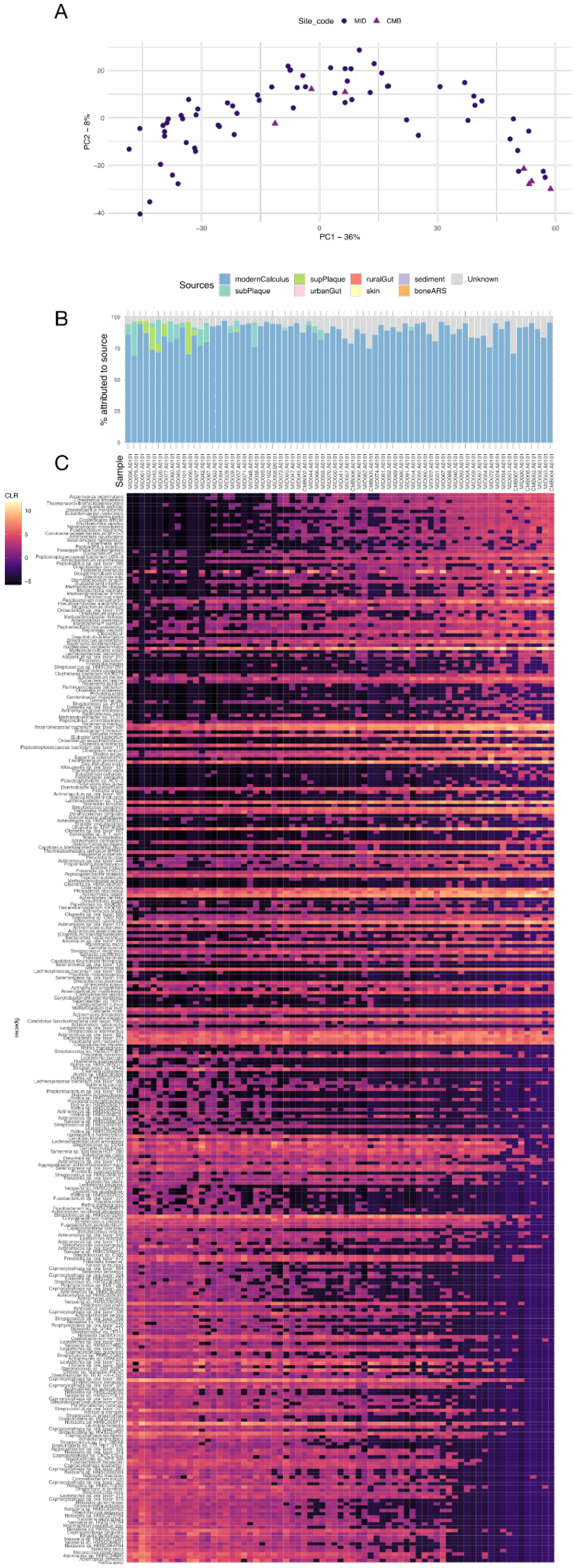

Figure S5. Species gradient in Middenbeemster (MID) and Convento de los Mercedarios de Burtzeña (CMB) calculus samples profiled with MALT. The sample order of plots B and C follows the order of points along PC1 in the PCA shown in panel A. **A.** PCA of read-based species counts result in a horseshoe pattern PCA plot. **B.** SourceTracker results indicate that the samples with the most negative PC1 loadings have higher proportions of species found in supra- and subgingival dental plaque than the other samples. **C.** Heat map showing the CLR-transformed abundance of species present at > 0.01% abundance. A gradient of taxa in samples from one end of the PCA to the other end can be traced from the upper right corner to the lower left corner. Samples with the most negative PC1 loadings have higher proportions of early-colonizer, aerobic and facultative taxa, and lower proportions of late-colonizer, anaerobic taxa than samples with the most positive PC1 loadings, consistent with a higher source contribution of plaque seen in panel B.

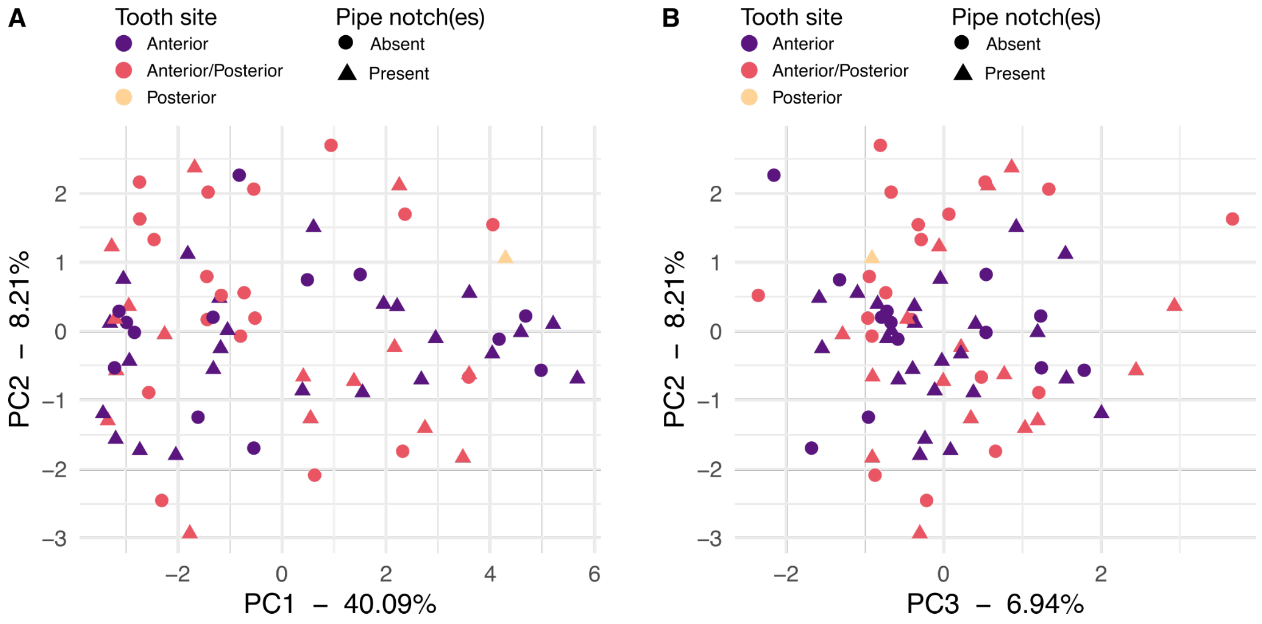

Figure S6. PCA of Middenbeemster and Convento de los Mercedarios de Burtzeña samples, colored by the location of teeth off of which calculus was collected. Most samples are pooled off multiple teeth, and nearly half are pooled from both anterior and posterior teeth. **A.** Plot showing PC1 and PC2. **B.** Plot showing PC3 and PC2.

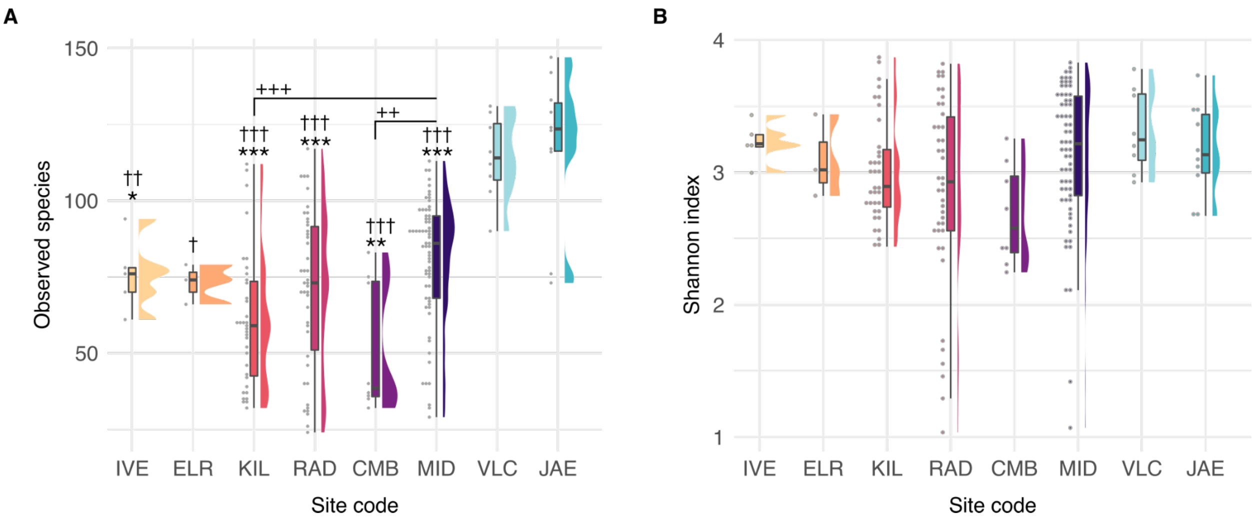

Figure S7. Alpha-diversity of samples grouped by site. A. Number of species. B. Shannon index. *** $p < 0.001$, ** $p < 0.01$, * $p < 0.05$ compared to JAE. ††† $p < 0.001$, †† $p < 0.01$, † $p < 0.05$ compared to VLC. ++ $p < 0.01$, +++ $p < 0.001$ compared to MID. No significant differences in Shannon index were detected between sites. Site codes: **IVE** - Iglesia de la Virgen de la Estrella; **ELR** - El Raval; **KIL** - Kilteasheen; **RAD** - Radcliffe; **CMB** - Convento de los Mercedarios de Burtzeña; **MID** - Middenbeemster; **VLC** - Valencia; **JAE** - Jaen.

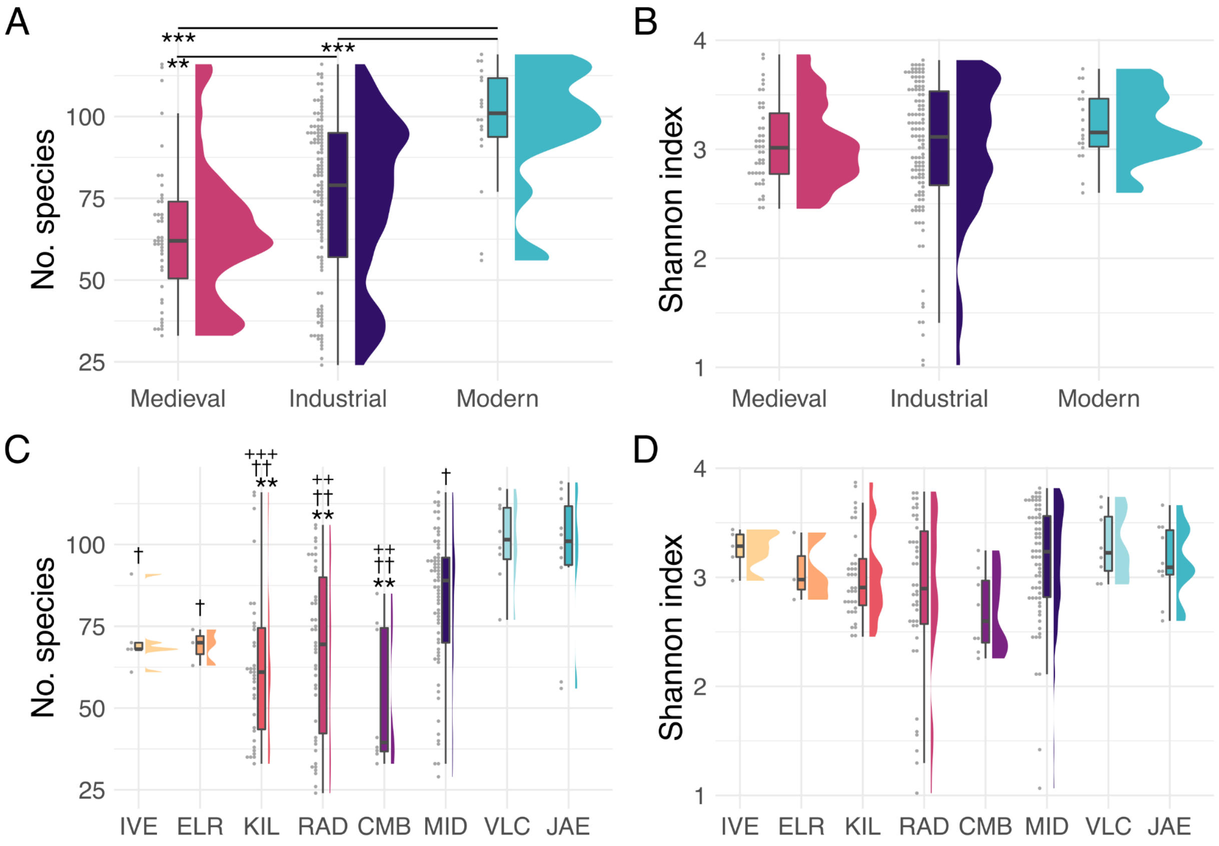

Figure S8. Alpha-diversity after sub-sampling all libraries to a maximum of 10M reads. **A.** Number of species grouped by time period. **B.** Shannon index grouped by time period. **C.** Number of species grouped by site. **D.** Shannon index grouped by site. *** $p < 0.001$, ** $p < 0.01$, * $p < 0.05$ compared to JAE. ††† $p < 0.001$, †† $p < 0.01$, † $p < 0.05$ compared to VLC. + $p < 0.05$, ++ $p < 0.01$, +++ $p < 0.001$ compared to MID. No significant differences in Shannon index were detected between time periods or sites. Site codes: **IVE** - Iglesia de la Virgen de la Estrella; **ELR** - El Raval; **KIL** - Kilteasheen; **RAD** - Radcliffe; **CMB** - Convento de los Mercedarios de Burtzeña; **MID** - Middenbeemster; **VLC** - Valencia; **JAE** - Jaen.

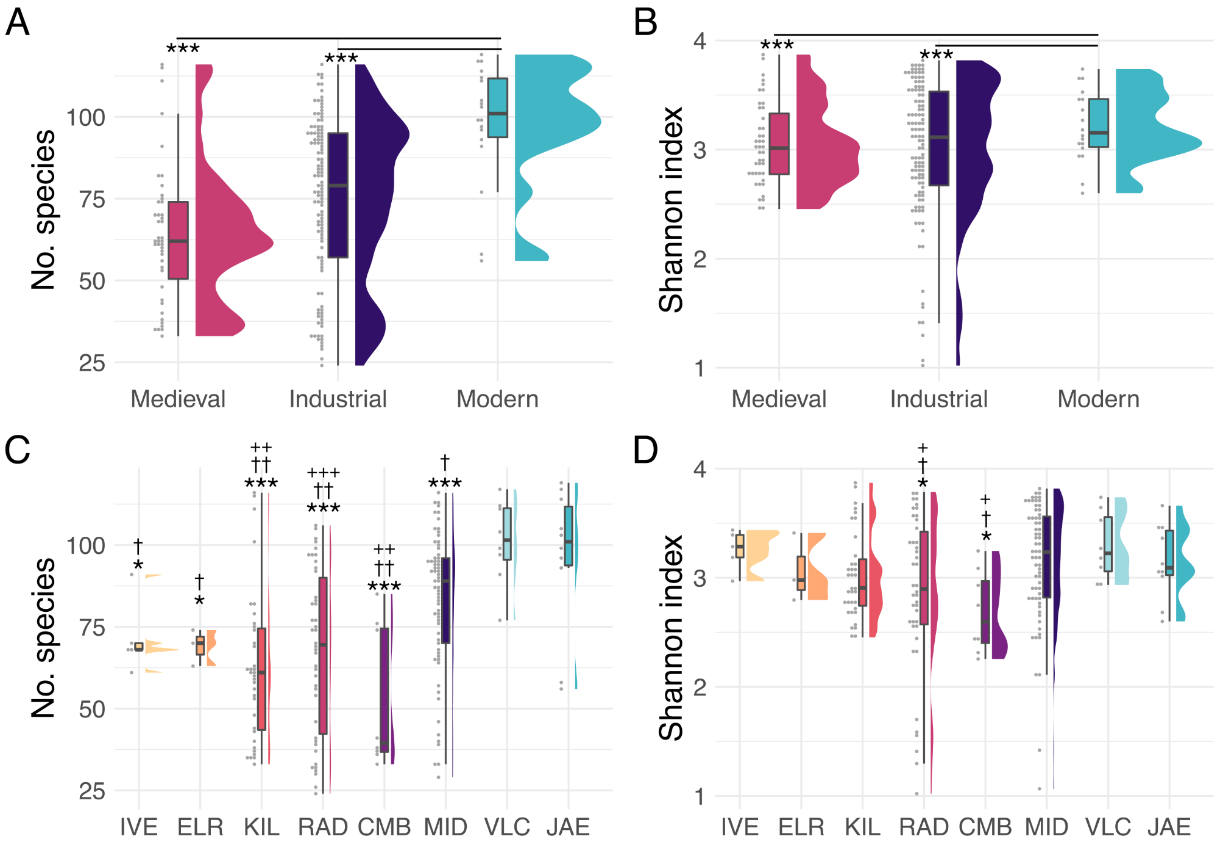

Figure S9. Alpha-diversity after sub-sampling all libraries for only reads 75 bp. **A.** Number of species grouped by time period. **B.** Shannon index grouped by time period. **C.** Number of species grouped by site. **D.** Shannon index grouped by site. *** $p < 0.001$, ** $p < 0.01$, * $p < 0.05$ compared to JAE. ††† $p < 0.001$, †† $p < 0.01$, † $p < 0.05$ compared to VLC. + $p < 0.05$, ++ $p < 0.01$, +++ $p < 0.001$ compared to MID. No significant differences in Shannon index were detected between time periods. Site codes: **IVE** - Iglesia de la Virgen de la Estrella; **ELR** - El Raval; **KIL** - Kilteasheen; **RAD** - Radcliffe; **CMB** - Convento de los Mercedarios de Burtzeña; **MID** - Middenbeemster; **VLC** - Valencia; **JAE** - Jaen.

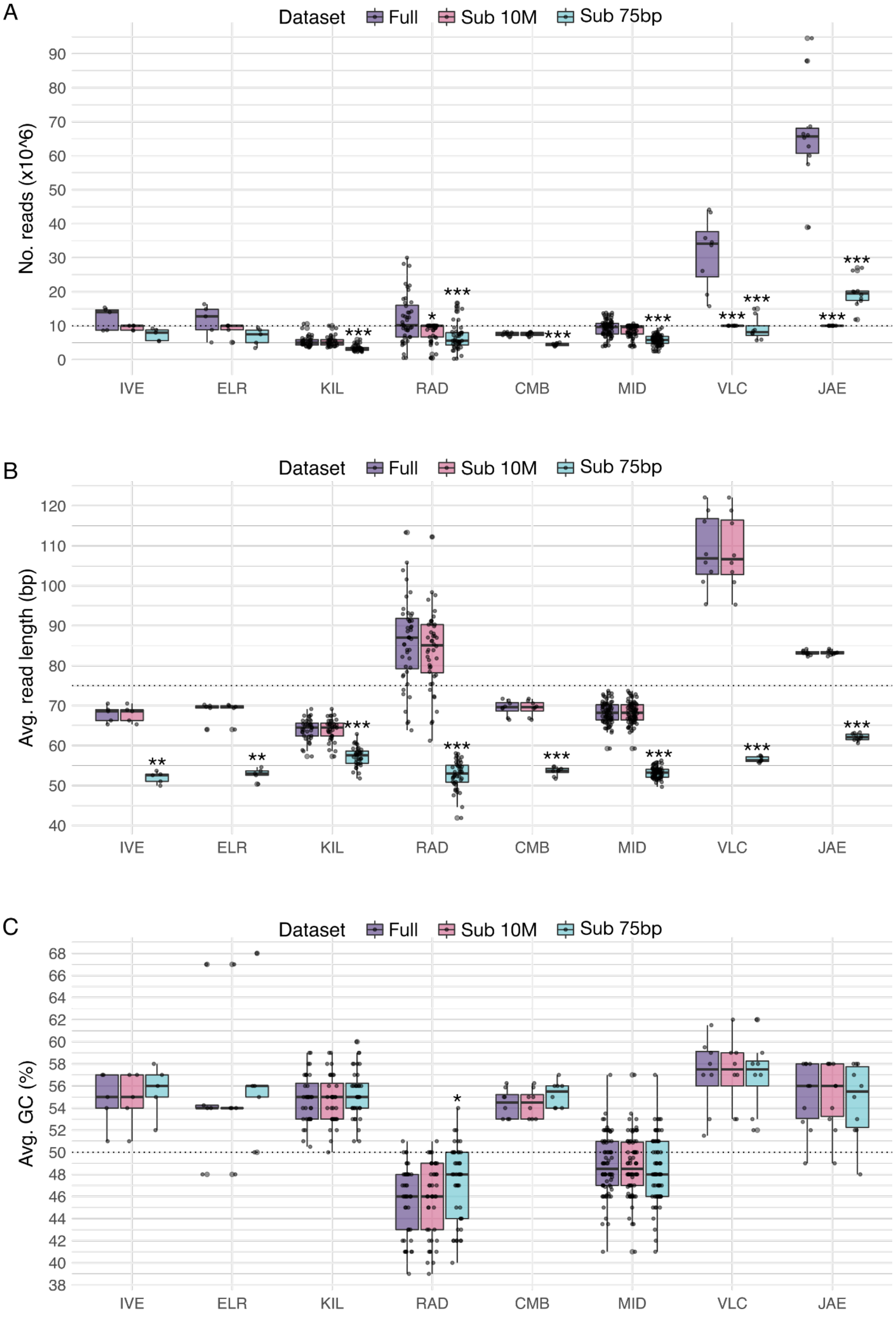

Figure S10. Read characteristics for full and sub-sampled libraries. **A.** Total number of reads. **B.** Average read length (bp). **C.** Average GC content (%). **Sub 10M** - libraries subsampled to include no more than 10 million reads. **Sub 75bp** - libraries subsampled to include only reads ≤ 75bp in length. *** $p < 0.001$, ** $p < 0.01$, * $p < 0.05$ compared to the full dataset.

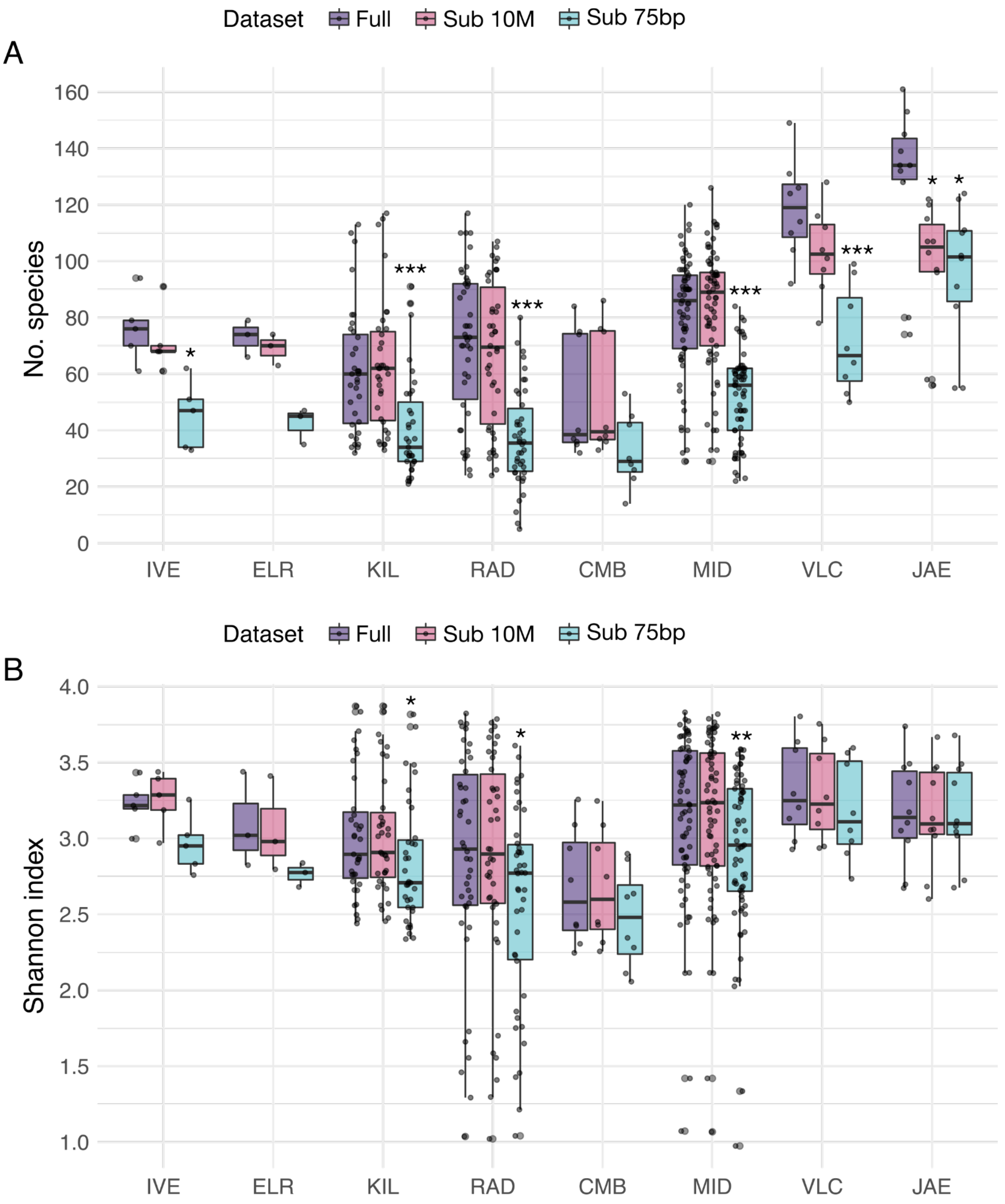

Figure S11. Diversity indices for full and sub-sampled libraries. **A.** Number of species. **B.** Shannon index. *** p < 0.001, ** p < 0.01, * p < 0.05 compared to the full dataset. **Sub 10M** - libraries subsampled to include no more than 10 million reads. **Sub 75bp** - libraries subsampled to include only reads ≤ 75bp in length.

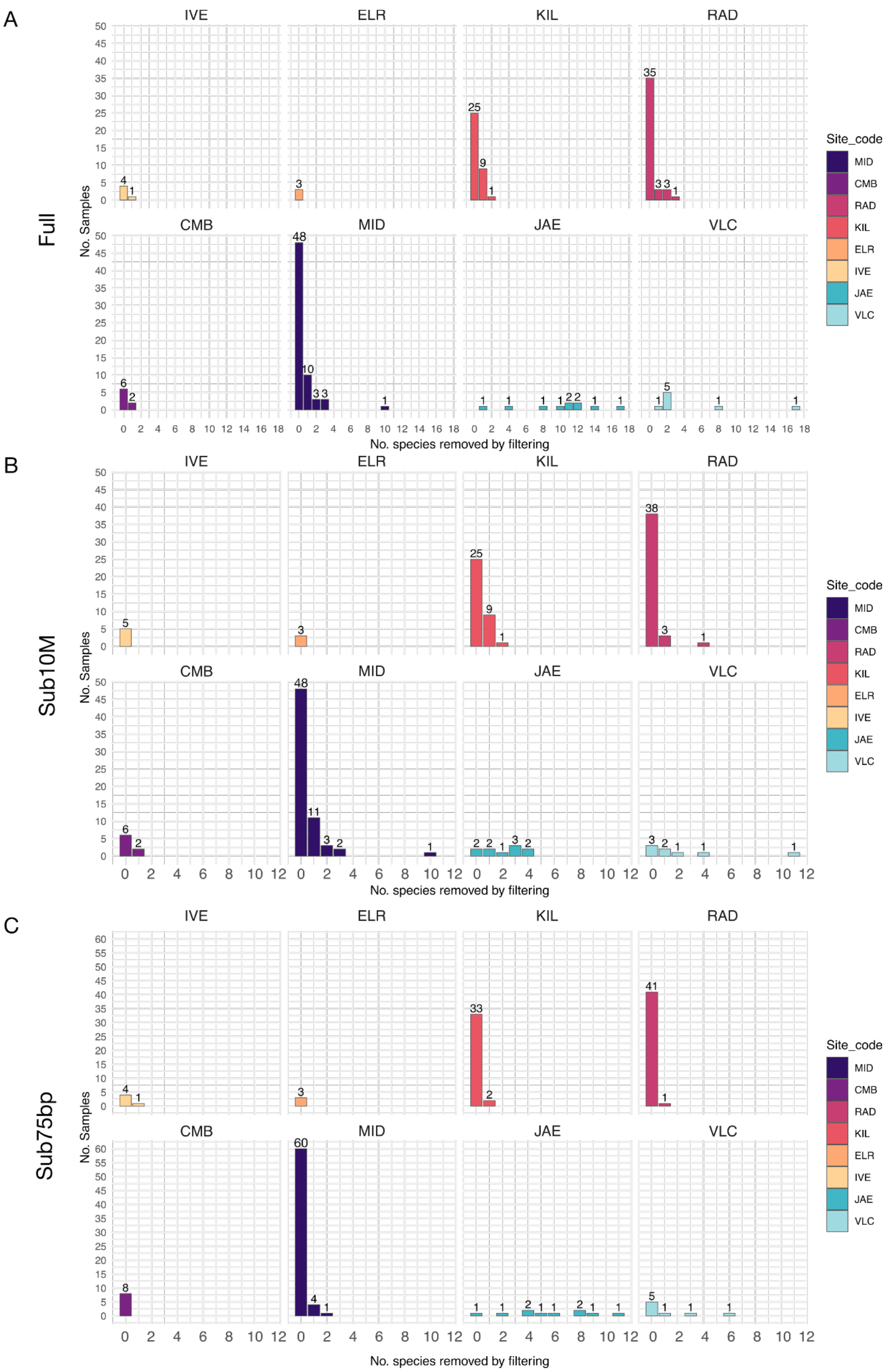

Figure S12. Species removed per sample by filtering the MetaPhlAn3 species tables to remove all species present at < 0.001% abundance. The total number of samples is shown above each bar. **A.** Full data set. **B.** Data set subsetted for no more than 10M reads per sample. **C.** Data set subsetted to include only reads 75bp or shorter.

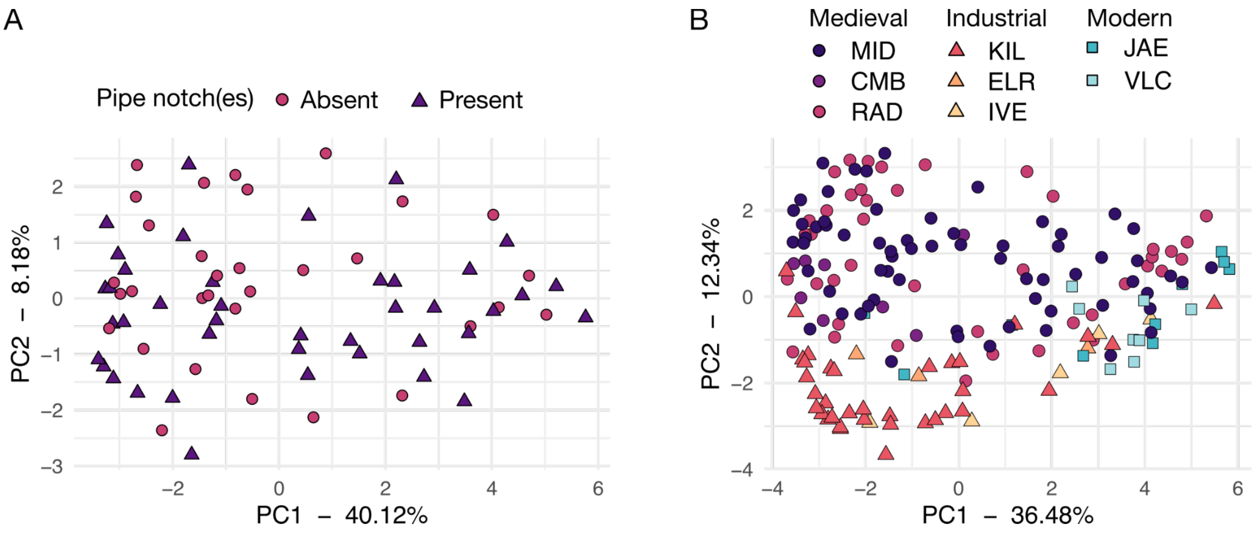

Figure S13. PCAs based on unfiltered MetaPhlAn3 data tables. **A.** Middenbeemster and Convento de los Mercedarios de Burtzeña samples, colored and shaped by presence of pipe notches. Contrast with main text Figure 2. **B.** All data sets, colored and shaped by site. Contrast with main text Figure 5 and Supplemental Figure S14.

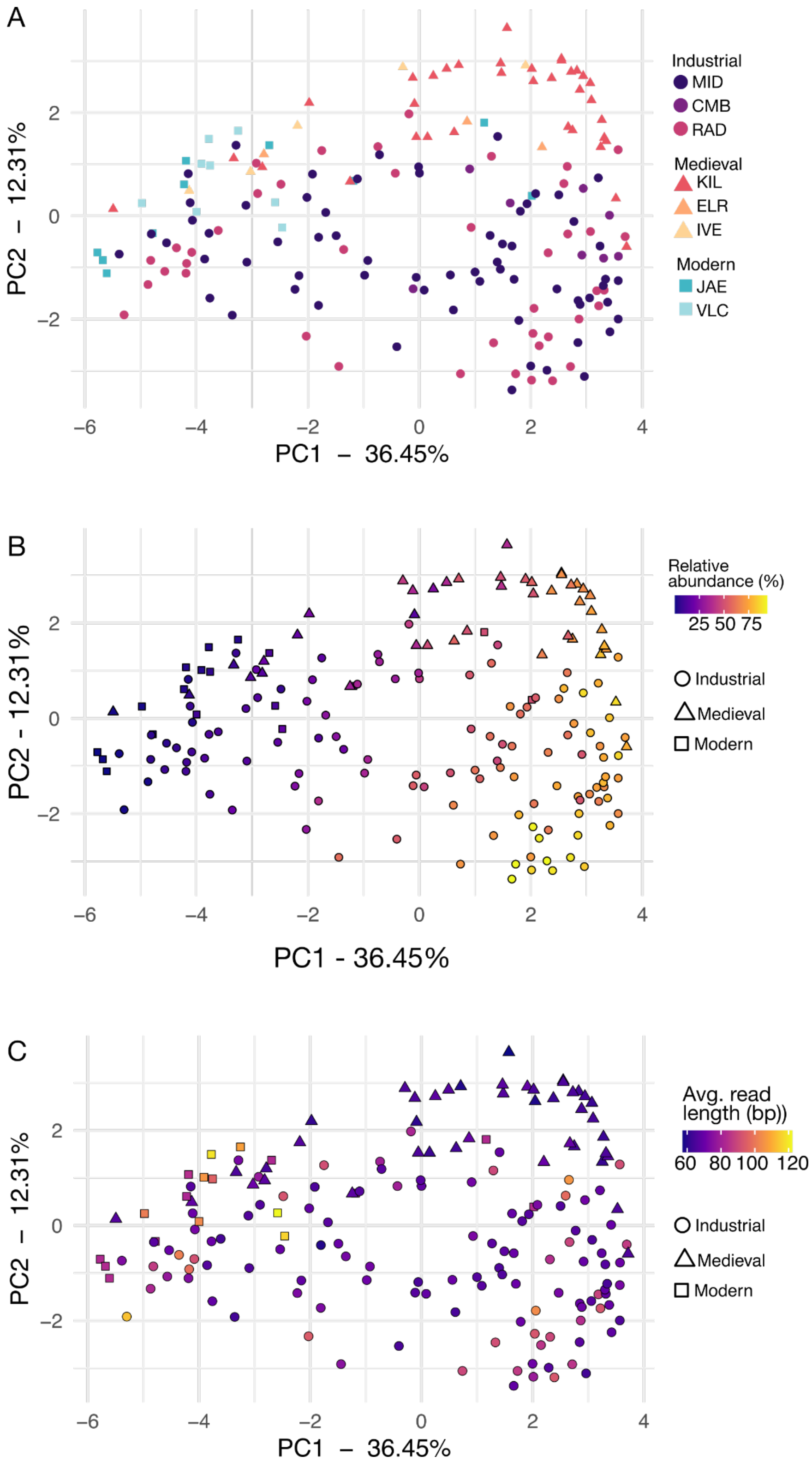

Figure S14. Beta-diversity PCA plot of calculus samples from pre- and post-introduction of tobacco to Europe. Shapes in both indicate time period. These are the same plot as main text Figure 5 but colored by **A.** Site. **B.** Relative abundance of the 10 species with strongest positive PC1 loadings, all of which are anaerobic species found late in dental biofilm development. See Supplemental table SXX4. **C.** Average read length per sample.

Table S4. Top 10 species with strongest loadings in PC1 in the PCA with pre- and post-tobacco introduction to Europe samples (Figure 5, Supplemental Figure S14).

PC1 direction	Species*	Aerotolerance
Positive	*Eubacterium minutum*	Anaerobic
Positive	Anaerolineaceae bacterium oral taxon 439	Anaerobic
Positive	*Methanobrevibacter oralis*	Anaerobic
Positive	*Desulfobulbus oralis*	Anaerobic
Positive	*Fretibacterium fastidiosum*	Anaerobic
Positive	*Desulfomicrobium orale*	Anaerobic
Positive	*Eubacterium saphenum*	Anaerobic
Positive	*Peptostreptococcaceae* bacterium oral taxon 113	Unclear
Positive	*Tannerella forsythia*	Anaerobic
Positive	*Treponema socranskii*	Anaerobic
Negative	*Streptococcus sanguinis*	Facultative
Negative	*Lautropia mirabilis*	Facultative
Negative	*Neisseria sicca*	Aerobic
Negative	*Neisseria mucosa*	Aerobic
Negative	*Ottowia* sp oral taxon 894	Unclear
Negative	*Neisseria elongata*	Aerobic
Negative	*Capnocytophaga sputigena*	Facultative
Negative	*Capnocytophaga gingivalis*	Facultative
Negative	*Rothia aeria*	Aerobic
Negative	*Streptococcus oralis*	Facultative

* Species are ordered from strongest to weakest loading.

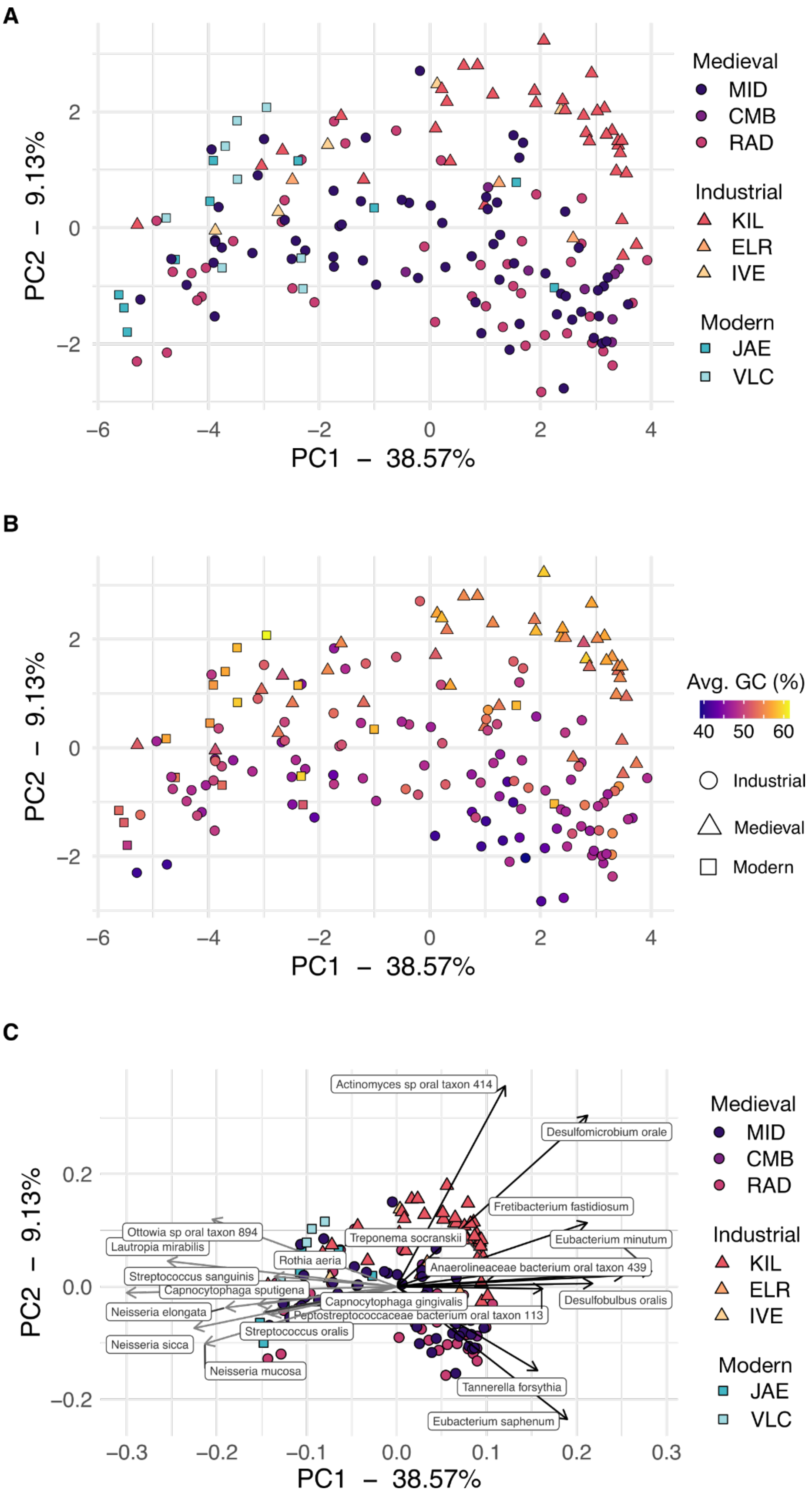

Figure S15. PCA of the species table excluding *Methanobrevibacter oralis*. Removal of this species only slightly alters the relationship of samples in the PCA. **A.** PCA with points colored by site and shaped by time period. **B.** Same as A with points colored by sample average GC content. **C.** Same as A with a biplot showing the top 10 species with strongest PC1 positive and negative loadings. Site codes: **MID** - Middenbeemster, **CMB** - Convento de los Mercedarios de Burtzeña, **RAD** - Radcliffe, **KIL** - Kilteasheen, **ELR** - El Raval, **IVE** - Iglesia de la Virgen de la Estrella, **JAE** - Jaen, **VLC** - Valencia.

References

1. R. de Jong, M. E. Mit, G. J. Pielage, A. J. Haartsen, “Nominatiedossier (nederlandse versie): droogmakerij de Beemster aan de hand waarvan de UNESCO droogmakerij de Beemster op 1 december 1999 op de werelderfgoedlijst heeft geplaatst” (Netherlands Department for Conservation, Zeist, 1988).

2. V. S. E. Falger, C. A. Beemsterboer-Köhne, A. J. Kölker, *Nieuwe Kroniek van de Beemster* (Serendipity, 2012).

3. D. Aten, *et al.*, *400 jaar Beemster 1612-2012* (Wormerveer: Stichting Uitgeverij Noord-Holland, 2012).

4. H. Leroux, “The use of dental nonmetric traits for intracemetery kinship analysis and cemetery structure analysis from the site of Middenbeemster, the Netherlands,” University of Leiden. (2012) (January 13, 2022).

5. J. K. Veldman, “Non-metric traits. An assessment of cranial and post-cranial non-metric traits in the skeletal assemblage from the 17th-19th century churchyard of middenbeemster, the Netherlands,” University of Leiden. (2013) (January 13, 2022).

6. J. E. Buikstra, D. H. Ubelaker, Standards for Data Collection from Human Skeletal Remains. *The Quarterly Review of Biology* **70**, 539–540 (1995).

7. Workshop of European Anthropologists, Recommendations for age and sex diagnoses of skeletons. *J. Hum. Evol.* **9**, 517–549 (1980).

8. G. J. Maat, Diet and age-at-death determinations from molar attrition. *A review related to the Low Countries. J. Forensic Odonto-Stomatol* **19**, 18–21 (2001).

9. C. O. Lovejoy, R. S. Meindl, T. R. Pryzbeck, R. P. Mensforth, Chronological metamorphosis of the auricular surface of the ilium: a new method for the determination of adult skeletal age at death. *Am. J. Phys. Anthropol.* **68**, 15–28 (1985).

10. M. Y. Işcan, S. R. Loth, Determination of age from the sternal rib in white females: a test of the phase method. *J. Forensic Sci.* **31**, 990–999 (1986).

11. S. Brooks, J. M. Suchey, Skeletal age determination based on the os pubis: A comparison of the Acsádi-Nemeskéri and Suchey-Brooks methods. *Hum. Evol.* **5**, 227–238 (1990).

12. J. L. Buckberry, A. T. Chamberlain, Age estimation from the auricular surface of the ilium: a revised method. *Am. J. Phys. Anthropol.* **119**, 231–239 (2002).

13. E. Domínguez Ballesteros, L. S. Zufiaurre, M. I. G. Collado, El monasterio mercedario de Burtzeña. *Kobie. Paleoantropología*, 185–198 (2018).

14. M. I. García-Collado, E. D. Ballesteros, L. S. Zufiaurre, Estudio osteoarqueológico de la población humana enterrada en el Convento Mercedario de Burtzeña (Barakaldo, Bizkaia),

finales s. XVI--principios s. XIX. *Kobie Paleoantropología* **36**, 199–222 (2018).

15. T. W. Phenice, A newly developed visual method of sexing the os pubis. *Am. J. Phys. Anthropol.* **30**, 297–301 (1969).

16. N. V. Passalacqua, Forensic Age-at-Death Estimation from the Human Sacrum. *Journal of Forensic Sciences* **54**, 255–262 (2009).

17. J. T. Martí, J. R. O. Pérez, I. R. Gómez, M. A. E. Bebia, El cementerio mudéjar del Raval (Crevillent-Alicante). *AyTM* **16**, 179–216 (2009).

18. J. Trelis, J. R. Ortega, I. Reina, M. A. Esquembre, El Cementeri Mudèjar del Raval (Crevillent-Alacant). *Rella, La*, 213–252 (2010).

19. M. P. de Miguel Ibáñez, Anexo. Necrópolis mudéjar de Crevillent: estudio osteoarqueológico. *Lucentum*, 221–231 (2007).

20. I. Gately, *La Diva Nicotina: The Story of how Tobacco Seduced the World* (Scribner, 2001).

21. M. Norton, *Sacred Gifts, Profane, Pleasures: A History of Tobacco and Chocolate in the Atlantic World* (Cornell University Press, 2008).

22. G. A. Brongers, *Nicotiana tabacum: the history of tobacco and tobacco smoking in the Netherlands* (Becht, 1964).

23. J. Goodman, *Tobacco in history: The cultures of dependence* (Routledge, 1993).

24. R. D. Stam, “Vergeten glorie: De economische ontwikkeling van de Nederlandse kleipijpennijverheid in de 17e en 18e eeuw, met speciale aandacht voor de export,” Universiteit Utrecht. (2019).

25. A. Hakvoort, “De begravingen bij de Keyserkerk te Middenbeemster” (HOLLANDIA archeologen, 2013).

26. M. L. Schabbink, “Een Beemster Poldermolen. Archaeologisch onderzoek rond de Draaioorder molengang in Zuidoostbeemster” (Noord-Hollandse Archaeologisch Publicaties - 9. Huis Van Hilde Archaeologiecentrum Noord-Holland: Castricum. , 2020).

27. S. A. Inskip, L. Zachary, M. Serrano Ruber, M. L. P. Hoogland, Pipe Smoking and Oral Health in Males from the Netherlands during the 18th-19th century. *Post Medieval Archaeology*.

28. J. B. de Heredia Bercero, N. M. i. Alaix, M. Soberón, Production and Trade of short stemmed Clay Pipes Found in Barcelona between the Seventeenth and the Nineteenth Century. *Journal of the Academie Internationale De La Pipe* **5** (2012).

29. C. Cortes Bárcena, Dutch and English clay pipes found in Santander (Cantabria, Spain). *Journal of the Académie Internationale de la Pipe* **6**, 83–85 (2013).

30. J. A. Fellows Yates, *et al.*, The evolution and changing ecology of the African hominid oral microbiome. *Proc. Natl. Acad. Sci. U. S. A.* **118** (2021).

31. D. H. Huson, *et al.*, MEGAN Community Edition - Interactive Exploration and Analysis of

Large-Scale Microbiome Sequencing Data. *PLoS Comput. Biol.* **12**, e1004957 (2016).

32. N. M. Davis, D. M. Proctor, S. P. Holmes, D. A. Relman, B. J. Callahan, Simple statistical identification and removal of contaminant sequences in marker-gene and metagenomics data. *Microbiome* **6**, 226 (2018).

33. D. Knights, *et al.*, Bayesian community-wide culture-independent microbial source tracking. *Nat. Methods* **8**, 761–763 (2011).

34. I. M. Velsko, *et al.*, Microbial differences between dental plaque and historic dental calculus are related to oral biofilm maturation stage. *Microbiome* **7**, 102 (2019).
